# Inhibition of a NF-κB/Diap1 Pathway by PGRP-LF Is Required for Proper Apoptosis during *Drosophila* Development

**DOI:** 10.1371/journal.pgen.1006569

**Published:** 2017-01-13

**Authors:** Raphael Tavignot, Delphine Chaduli, Fatoumata Djitte, Bernard Charroux, Julien Royet

**Affiliations:** Aix Marseille Univ, CNRS, IBDM, Marseille, France; Harvard Medical School, Howard Hughes Medical Institute, UNITED STATES

## Abstract

NF-κB pathways are key signaling cascades of the *Drosophila* innate immune response. One of them, the Immune Deficiency (IMD) pathway, is under a very tight negative control. Although molecular brakes exist at each step of this signaling module from ligand availability to transcriptional regulation, it remains unknown whether repressors act in the same cells or tissues and if not, what is rationale behind this spatial specificity. We show here that the negative regulator of IMD pathway PGRP-LF is epressed in ectodermal derivatives. We provide evidence that, in the absence of any immune elicitor, PGRP-LF loss-of-function mutants, display a constitutive NF-κB/IMD activation specifically in ectodermal tissues leading to genitalia and tergite malformations. In agreement with previous data showing that proper development of these structures requires induction of apoptosis, we show that ectopic activation of NF-κB/IMD signaling leads to apoptosis inhibition in both genitalia and tergite primordia. We demonstrate that NF-κB/IMD signaling antagonizes apoptosis by up-regulating expression of the anti-apoptotic protein Diap1. Altogether these results show that, in the complete absence of infection, the negative regulation of NF-κB/IMD pathway by PGRP-LF is crucial to ensure proper induction of apoptosis and consequently normal fly development. These results highlight that IMD pathway regulation is controlled independently in different tissues, probably reflecting the different roles of this signaling cascade in both developmental and immune processes.

## Author Summary

In multicellular organism such as mammals or insects, activation of innate immune responses occurs following detection of microbes by dedicated receptors called pattern recognition receptors. Such immune activation is taking place in immune competent tissue such as the skin, the digestive and respiratory epithelia and is under a tight negative control. Negative control is essential to finely adjust the duration and the intensity of the immune response to the level of infection. We found that the *Drosophila* innate immunity negative regulator PGRP-LF, is specifically expressed in non-immune tissues and plays an essential role during development, in absence of any infection. Lack of PGRP-LF function in these tissues inhibits apoptosis leading to incomplete genitalia rotation and tergite malformations. We show that such apoptosis inhibition results from the over expression of the negative regulator of apoptosis Diap1 specifically in PGRP-LF expressing cells. Our data highlight that proper negative regulation of immune signaling pathway in non-immune tissues is contributing to normal development and illustrate the growing evidence of the dual role of immune signaling pathway contribution to both immunity and in development processes.

## Introduction

In *Drosophila*, bacteria infection triggers NF-κB cascades (called Toll and IMD (Immune Deficiency)) leading to the production of immune effectors and regulators [1–3] [4]. This activation relies on the previous recognition of bacteria derived peptidoglycan (PGN) by host Peptidoglycan Recognition Protein (PGRP) family members. Recognition of Gram-positive PGN by circulating PGRP-SA triggers the maturation of the pro-Spätzle protein into an active ligand for the Toll membrane receptor [5]. The IMD pathway is triggered upon recognition of PGN by either transmembrane associated PGRP-LC or cytoplasmic PGRP-LE [5–10]. Receptor activation leads to the recruitment of the death-domain containing adapters IMD, FADD, and the caspase DREDD. Activated DREDD cleaves IMD, thus allowing its poly-ubiquitination that allows recruitment of the TAK1/TAB2 and the IRD5/Kenny kinase to the receptor complex [11]. These interactions ultimately lead to the nuclear translocation of the transcription factor Relish. In contrast to Toll signaling, IMD pathway activation after bacterial infection is transient and buffered by many repressors [12–14]. This tight control might reflect the essential role played by the IMD cascade in controlling antibacterial response in fly epithelia [4, 15]. Indeed, the constant contact between bacteria and epithelia requires the presence of immune tolerance mechanisms through which the epithelium copes with the continuous input from microbiota derived immune-activating signals [16–19]. The homeobox transcription factor Caudal was one of the first proteins identified as an IMD pathway antagonist [20]. Through its occupation of some IMD target promoters, Caudal blocks IMD-dependent transcription. Negative regulation is also mediated through protein turnover by ubiquitous factors that regulate protein stability of identified IMD pathway components (dUSP36, CYLD, DNR-1, Caspar) [21–23] [24–26]. Some of IMD pathway regulation is also taking place at the level of the PGRP-LC receptor itself and of its PGN ligand. PGRP-LC transcription is under the control of the steroid hormone ecdysone [27]. The number of PGRP-LC molecules at the membrane, depends on intracellular (Pirk) and membrane associated (nonaspanins TM9SF2 and TM9SF4) proteins that by sequestering PGRP-LC in the cytoplasm prevent its localisation at the membrane [16, 28–31]. Another member of the PGRP family, PGRP-LF, antagonizes IMD pathway activation [32]. This transmembrane protein which has no intracytoplasmic tail but has two occluded PGRP domains is unable to bind PGN [32, 33]. Plasma resonance data show that by interacting with PGRP-LC ectodomain, PGRP-LF prevents constant activation of the IMD pathway even in the absence of bacteria [34]. IMD pathway tuning is also mediated through the modulation of ligand availability, via a family of extracellular enzymes called amidases, which degrade PGN into non-stimulatory fragments [17, 18, 35–40]. While the inhibition provided by these regulators appears to be constitutive, the negative regulation brought by amidase and by PIRK is the result of a negative feedback loop. As a result, these factors additively regulate the amplitude of the IMD response. While the detrimental effects of runaway inflammation in mammals are well established, the situation is less clear with regards to *Drosophila*. The absence of ubiquitous negative regulators leads to a reduced lifespan, which however cannot be ascribed specifically to the constitutive activation of the IMD pathway, as these regulators act upon multiple targets. Modulation of amidase levels causes deregulation of NF-κB activity in the gut, resulting in commensal dysbiosis, stem cell hyper-proliferation, epithelial dysplasia and eventually reduced life span [18, 40]. Importantly, these phenotypes can be partially rescued in germ-free conditions or by inactivating IMD pathway components demonstrating that they are direct consequences of IMD pathway stimulation by bacteria.

Although IMD pathway is controlled at multiple levels along the cascade, the rationale behind this complex and multilayered regulation is not fully understood. It remains to be known whether this regulation is tissue specific and if yes, what are the consequences of IMD activation in each tissue. Our results show that PGRP-LF is expressed in ectodermal derivatives some of them with no known immune function. Phenotypic analysis of a newly generated PGRP-LF protein null allele demonstrates that its function is required for the formation of various epidermal derived adult structures. We showed that PGRP-LF's role in controlling developmental process is, like for the immune ones, mediated by a repression of the IMD pathway. Our results demonstrate that, in contrast to what has been shown in other tissues, IMD pathway activation in epidermal structures is blocking rather than triggering apoptosis. By preventing such IMD mediated anti-apoptotic signal, PGRP-LF is allowing normal development to take place. These results speak for the importance of a tissue specific regulation of NF-κB pathway in flies and for a strong link between immune and developmental processes.

## Results

### PGRP-LF is expressed in ectodermal derivatives

**Fig 1:**
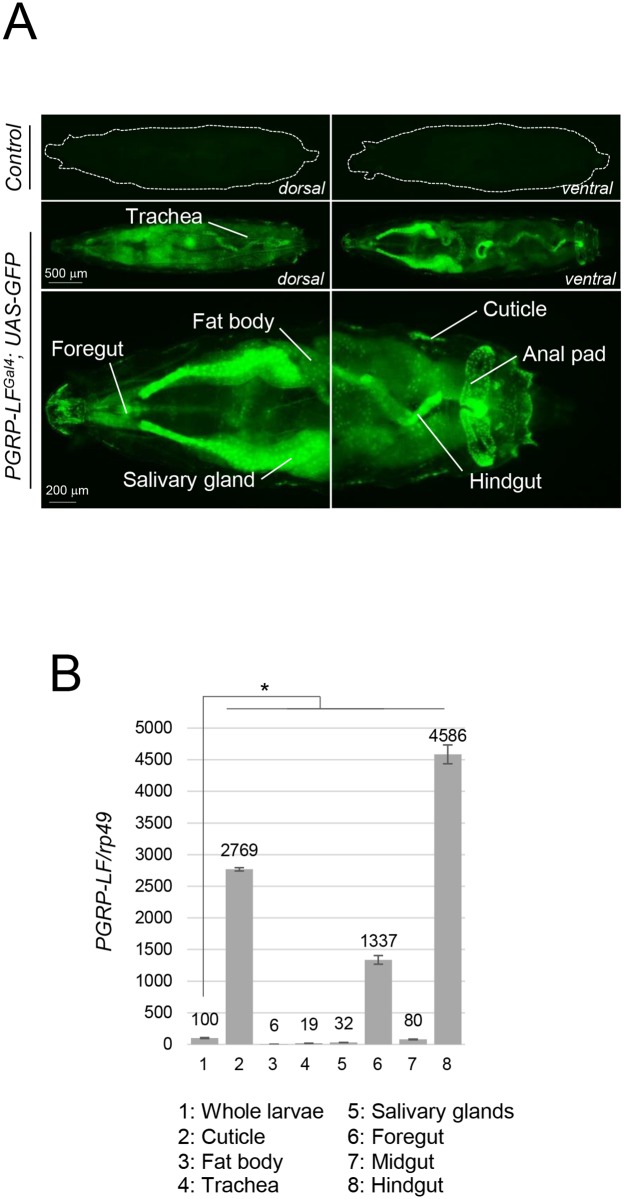
PGRP-LF is mostly expressed in ectodermal derivatives. (A) *PGRP-LF*^*Gal4*^, *UAS-nlsGFP* larvae showing GFP expression in salivary glands, foregut, hindgut and cuticle and to a lesser extend in fat body. *UAS-nlsGFP* larvae in a wild-type genetic background is shown as a negative control. (B) Relative *PGRP-LF mRNA expression* in third instar larvae dissected tissues. mRNA level in whole larvae was set to 100 and values obtained with dissected tissues were expressed as a fold of this value. For (B) histograms correspond to the mean value ± SD of three independent experiments. Values indicated by symbols (*) are statistically significant (t-test, p < 0.05). ns: not significantly different.

To reveal the expression pattern of the PGRP-LF gene, we generated reporter lines (later named *PGRP-LF*^*Gal4*^) in which 1.4 Kb of genomic DNA 5’ of the PGRP-LF coding region (corresponding to the PGRP-LC/PGRP-LF intergenic region, S1A Fig) was cloned upstream of Gal4 coding sequences. *PGRP-LF*^*Gal4*^, *UAS-GFP* larvae showed fluorescence in most larval ectodermal derivatives such as the cuticle, the salivary glands, the fore and hindguts and to a lesser extend, the trachea (Fig 1A and S2A Fig). mRNAs quantification confirmed the strong enrichment of *PGRP-LF* transcripts in ectodermal tissues (especially the cuticle, the foregut and the hindgut) and low levels in the fat body and in the midgut (when compared to foregut and hindgut), in accordance with FlyAtlas data (Fig 1B and S2B and S2C Fig). This was unexpected since these mesodermal derivatives are the main immune tissues in which bacteria infection is triggering IMD pathway activation. To elucidate the role of PGRP-LF in ectodermal tissues, we generated a novel *PGRP-LF* allele called *PGRP-LF*^*KO*^ (S1A Fig). Indeed, detailed molecular characterization demonstrated that the previously *PGRP-LF*^*200*^ allele is not molecularly null for PGRP-LF (S1B Fig). In addition, it retains P-element sequences 5' of the PGRP-LF ORF that might interfere with neighboring loci that code for proteins putatively interacting with PGRP-LF (PGRP-LC, PGRP-LA) (S1A Fig). The new *PGRP-LF*^*KO*^ allele obtained by homologous recombination, completely abolished PGRP-LF mRNA expression without affecting the transcription of the neighboring *UGP* gene (S1B and S3A Figs). The increased PGPR-LC transcription detected in *PGRP-LF*^*KO*^ flies is secondary to IMD pathway activation since it was reduced in IMD pathway mutant backgrounds (S3B Fig).

### PGRP-LF prevents constitutive NF-κB activation in ectodermal derivatives of axenic animals

**Fig 2:**
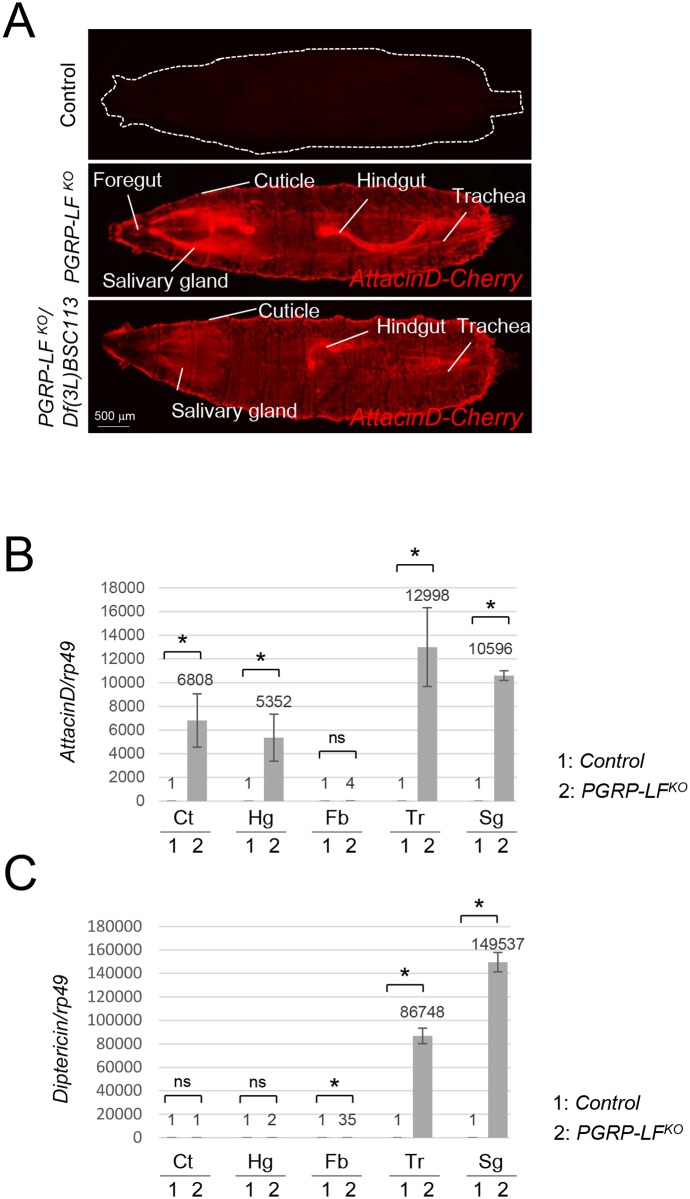
PGRP-LF inactivation triggers AMP ectopic expression in ectoderm derivatives. (A) AMPs ectopic expression in *PGRP-LF* mutant larvae (either *PGRP-LF*^*KO*^ or *PGRP-LF*^*KO*^*/Df(3L)BSC113*). *AttacinD-Cherry; PGRP-LF*^*KO*^ third instar larvae. AttacinD-Cherry expression is detected in trachea, hindgut, trachea, cuticle and salivary glands. *AttacinD-Cherry* larvae in a wild-type genetic background is shown as negative control. (B, C) Relative mRNA levels of *AttacinD (B)* and *Diptericin (C)* in third instar larvae tissues. mRNA level in control larvae was set to 1, and values obtained with dissected tissues were expressed as a fold of this value. Ct: cuticle, Hg: hindgut, Fb: fat body, Tr: trachea, Sg: salivary glands. For (A) and (B) histograms correspond to the mean value ± SD of three independent experiments. Values indicated by symbols (*) are statistically significant (t-test, p < 0.05). ns: not significantly different.

**Fig 3:**
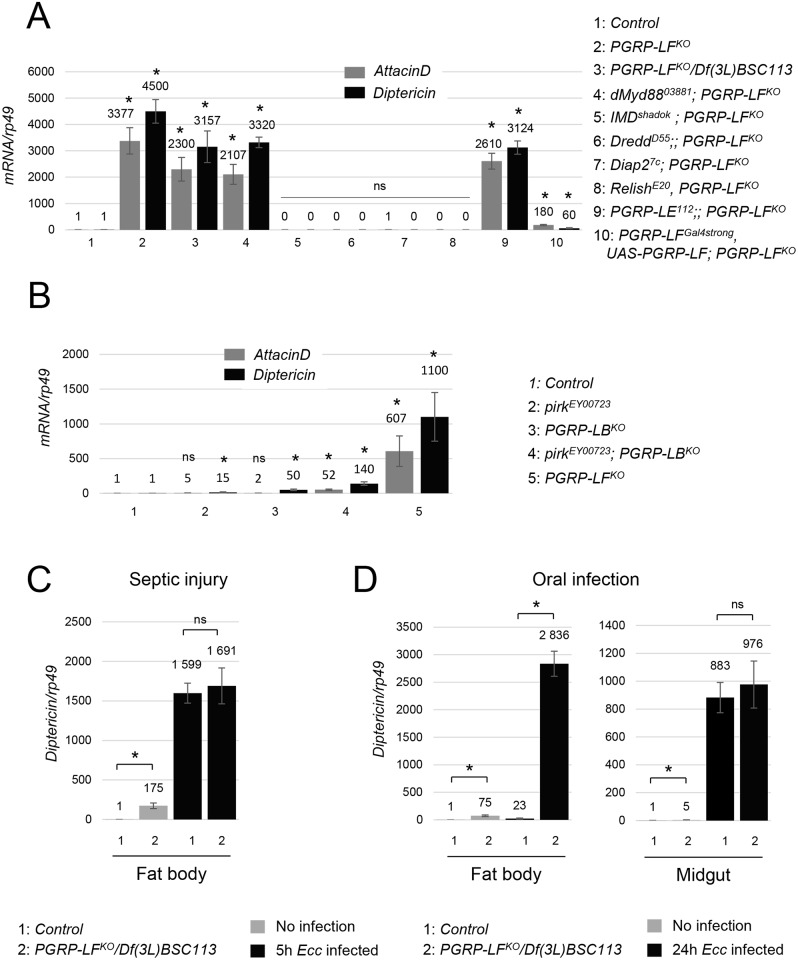
AMP expression in infected *PGRP-LF* mutants. (A) Overexpression of *AttacinD* and *Diptericin* mRNAs in *PGRP-LF* mutant larvae (either *PGRP-LF*^*KO*^ or *PGRP-LF*^*KO*^*/Df(3L)BSC113*) requires a functional PGRP-LC/IMD cascade. Inactivation of *IMD*, *Dredd*, *Diap2* and *Relish*, but not *dMyd88* or *PGRP-LE*, completely suppresses both *AttacinD* and *Diptericin* ectopic expression in *PGRP-LF* mutants. Expression of *UAS-PGRP-LF* under the control of *PGRP-LF*^*Gal4strong*^ suppresses the ectopic expression of AMPs observed in *PGRP-LF* mutants (B) Ectopic activation of AMP is not detected in mutants for other IMD pathway negative regulators such as *Pirk* or *PGRP-LB*. (C) IMD pathway activation, monitored by *Diptericin* expression, 5h after septic infection with *Ecc*. Although *Diptericin* is constitutively expressed at higher levels in uninfected *PGRP-LF* mutants (*PGRP-LF*^*KO*^*/Df(3L)BSC113*) than in wild-type, *Diptericin* mRNA levels are similar in fat body of wild-type and *PGRP-LF* mutant flies infected by septic injury. (D) IMD pathway activation, monitored by *Diptericin* expression, 24h after *Ecc* oral infection. While PGRP-LF inactivation does not modify IMD pathway inducibility in the midgut of *Ecc* orally infected flies, it does so in the fat body. For (A), (B), (C) and (D) mRNA level in controls was set to 1, and values obtained with indicated genotypes were expressed as a fold of this value. For (A) (B) (C) and (D) histograms correspond to the mean value ± SD of three independent experiments. Values indicated by symbols (*) are statistically significant (t-test, p < 0.05). ns: not significantly different.

Although previous results show that PGRP-LF negatively regulates the IMD pathway, the tissue specificity of PGRP-LF effects remain unknown [33]. We therefore compared the transcription levels of three IMD pathway target genes, *AttacinD*, *Drosomycin* and *Diptericin*, in wild-type and *PGRP-LF*^*KO*^ mutant flies. *PGRP-LF*^*KO*^ *or PGRP-LF*^*KO/*^*Df(3L)BSC113* flies showed a strong constitutive expression of *AttacinD* in most ectodermal larval derivatives (Fig 2A and 2B). Although *Diptericin* mRNA levels were up-regulated in the trachea and the salivary glands of *PGRP-LF* mutants, they were not in the cuticle and in the hindgut, demonstrating that IMD dependent antimicrobial peptide (AMP) genes transcription is differently regulated in different tissues (Fig 2C and S4A Fig). This is well illustrated with *Drosomycin* being only up-regulated in tracheal cells of *PGRP-LF*^*KO*^ larvae (S4A Fig). Although *Diptericin* is highly inducible by bacterial infection in fat body cells, a relatively mild increase of *Diptericin* mRNA was detected in this tissue when PGRP-LF was inactivated (Fig 2C). By combining *PGRP-LF*^*KO*^ mutants with *AttacinD-cherry* and *PGRP-LF*^*Gal4*^ /UAS-GFP reporters, we could show that tissues expressing PGRP-LF are the ones that display constitutive AMP expression upon PGRP-LF inactivation (S4B Fig). This tissue-autonomous expression indicated that AMP ectopic expression seen in *PGRP-LF* mutants was not due to global stress response of the larvae but rather to a direct consequence of IMD pathway activation in specific tissues. Epistatic experiments further showed that AMP ectopic expression observed in *PGRP-LF* mutants are due to PGRP-LC dependent IMD pathway activation. Indeed, AMP ectopic expression in PGRP-LF mutants was only moderately reduced by the functional inactivation of the intracytoplasmic receptor PGRP-LE and of the Toll signaling component dMyd88. In contrast, it was completely suppressed when IMD pathway components were inactivated (Fig 3A and S4C Fig). This is consistent with the fact that in tissues expressing PGRP-LF (trachea, hindgut, epidermis…), IMD pathway activation has been shown to rely on the upstream PGRP-LC transmembrane sensor [33]. It should be noted that the constitutive AMP expression was not observed in single mutants for other IMD pathway negative regulators such as *PGRP-LB* or *Pirk* although synergistic effects were observed (Fig 3B).

### PGRP-LF mutants are short-lived and susceptible to oral but not to systemic bacterial infection

**Fig 4:**
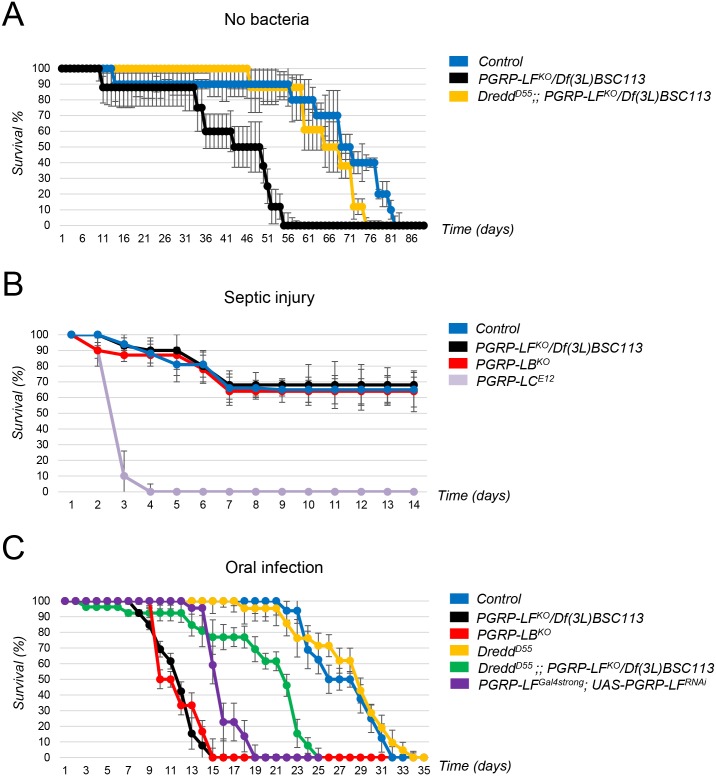
Effects of PGRP-LF inactivation on adult lifespan and ability to survive infection. (A) Survival analysis of control, *PGRP-LF*^*KO*^*/Df(3L)BSC113* and *Dredd*^*D55*^*; PGRP-LF*^*KO*^*/Df(3L)BSC113* mutants raised in axenic conditions. Ectopic activation of IMD pathway in *PGRP-LF* mutants reduces lifespan in axenic conditions. (B) Survival analysis of control, *PGRP-LF*^*KO*^*/Df(3L)BSC113*, *PGRP-LB*^*KO*^ and *PGRP-LC*^*E12*^ mutants after septic injury with *Ecc*. *PGRP-LF* mutants are as susceptible as controls but less susceptible than *PGRP-LC*^*E12*^ mutants. (C) Survival analysis of controls and mutants after oral infection with *Ecc*. *PGRP-LF* mutants (by either *PGRP-LF*^*KO*^*/Df(3L)BSC113 or PGRP-LF*^*Gal4strong*^*; UAS-PGRP-LF*^*RNAi*^*/UAS-Dicer2*) and *PGRP-LB*^*KO*^ are highly susceptible to *Ecc* oral infection. IMD pathway inactivation, via *Dredd*^*D55*^ mutation, partially suppresses *Ecc* induced lethality of *PGRP-LF* mutants. Survival curves are representative of at least five independent trials. Error bars indicate SD.

The above results demonstrated that PGRP-LF acts to prevent constitutive IMD pathway activation in the absence of bacteria. To appreciate the reason of such a regulation, we compared wild-type and *PGRP-LF* mutant fitness in the absence of bacteria. When flies were grown in axenic conditions, *PGRP-LF* mutants succumbed earlier than their wild-type siblings (Fig 4A). This premature death was largely suppressed by an absence of the caspase Dredd, demonstrating that the precocious lethality was mainly due to IMD constitutive activation. We then tested the ability of *PGRP-LF* mutant flies to mount an immune response to bacteria and to resist to them. While *PGRP-LF* adults showed, like *PGRP-LF* larvae, ectopic expression of AMP, their ability to trigger IMD pathway activation following *Ecc* septic injury was similar to that of wild-type controls (Fig 3C). Consistently, *PGRP-LF* mutant flies survived *Ecc* septic injury as well as control flies (Fig 4B). Interestingly, *Ecc* orally infected *PGRP-LF* mutants succumbed faster than controls with a kinetic close to that of *PGRP-LB* mutants (Fig 4C). Remarkably, *PGRP-LF* adult showed increased systemic AMP production when compared to controls, although local gut AMP production was normal (Fig 3D and S5 Fig). It is difficult to explain why *PGRP-LF* mutants show over-activation of IMD pathway in the fat body after *Ecc* oral infection but not after septic injury. The most likely reason is that the nature of the PGN that reaches PGRP-LC at the surface of the fat body cells is not the same whether it comes directly by pricking bacteria in the fly thorax or whether it translocates from the gut lumen. Indeed, PGN modifying enzymes, such as PGRP-SC and PGRP-LB, are differently expressed in the hemolymph and in the gut (Fig 4C). In any case, the reduced lifespan of *Ecc* orally infected mutants was partially rescue by inactivating *Dredd*. These results demonstrate that by preventing IMD pathway activation in the absence of infection, PGRP-LF prevents precocious death. They also show that although PGRP-LF does not affect the ability of the fly to respond to septic infection, it is required to prevent death following *Ecc* infection. AMP levels indicated that precocious lethality is probably due to IMD pathway over activation in fat body cells and not in enterocytes themselves.

### PGRP-LF mutant display developmental defects due to inappropriate IMD pathway activation

**Fig 5:**
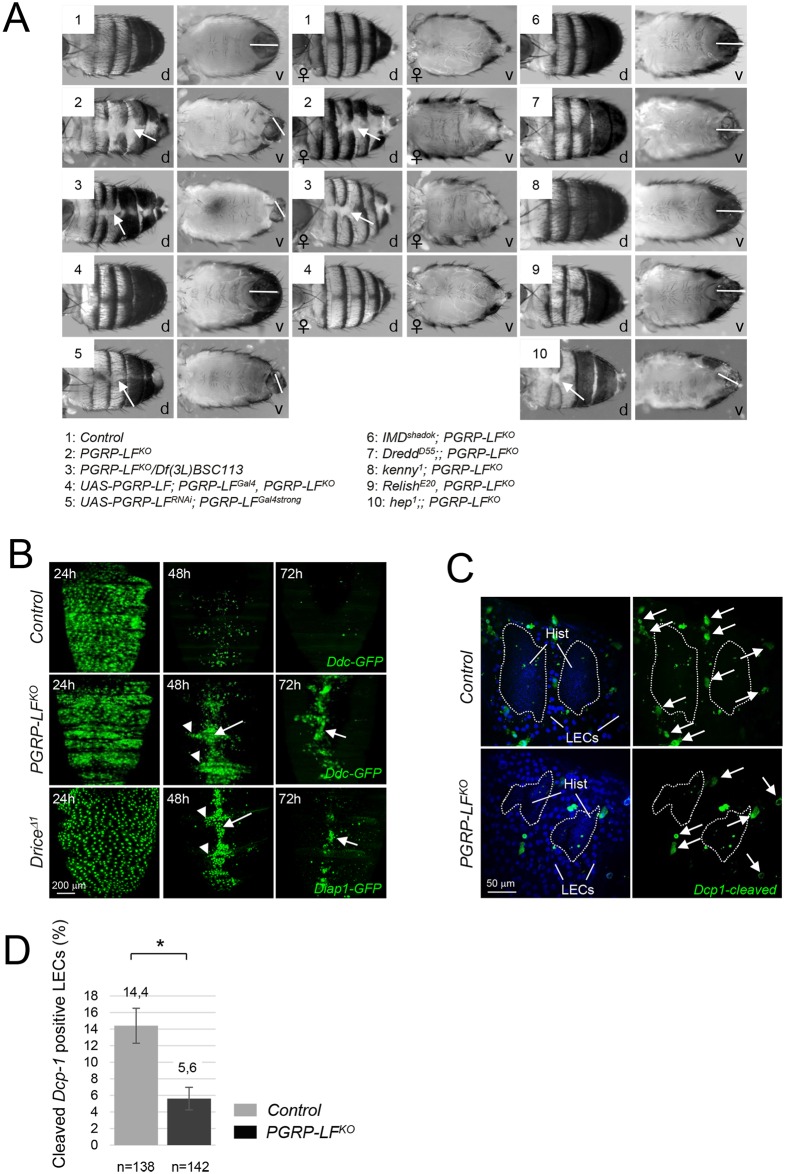
Adult *PGRP-LF* mutants display abdominal cuticle and male genitalia defects. (A) Dorsal (d) and ventral (v) views of male or female abdomens showing incomplete epidermis differentiation (white arrow) and abnormal male genitalia orientation (white bar). (B) Elimination of larval epidermal (LECs) cells is not taking place in *PGRP-LF* mutants. LECs are labeled with the *Ddc*^*Gal4*^ driver combined with UAS-GFP. LECs are prominently distributed along the surface of the dorsal abdomen of control pupae at 24 hours APF, but by 48 hours APF most of LECs have been eliminated. By 72 hours APF, only very few intact LECs remains. At 48h and 72h APF, *PGRP-LF*^*KO*^ and *Drice*^*Δ1*^ mutant pupae present many persistent LECs that accumulate along the midline of the abdomen (white arrow), and at segmental borders (arrowhead). (C) Defective LEC cell death in *PGRP-LF* mutant cuticle. Pictures show the lateral abdomen of 24h hours APF pupae stained with the anti-cleaved Dcp-1 antibody which labels caspase-activating LECs (arrows). The dashed lines indicate the boundary between the histoblasts and the LECs. (D) Quantification of dying LECs for 6 pupae 24h hours APF is shown in the histogram. For (D) histograms correspond to the mean value ± SD of six samples. Values indicated by symbols (*) are statistically significant (t-test, p < 0.05).

The *PGRP-LF*^*KO*^ allele is sub lethal with only some pupae (27% +/- 7 SD, n = 430) hatching as adults [33]. All adults and the dead pupae exhibited, with a range of severity, stereotypic malformations in the abdominal tergites with disruption in the joining of the cuticular plates along the dorsal midlines of the abdomen, exposing underlying soft tissue (Fig 5A). In addition, all *PGRP-LF*^*KO*^ male escapers displayed defects in male genitalia orientation (Fig 5A and S6B Fig). Theses phenotypes were completely rescued by expressing the wild-type *PGRP-LF* cDNA with the *PGRP-LF*^*Gal4*^ driver, demonstrating that they were indeed due to a lack of PGRP-LF protein in the ectodermal territories (Fig 5A). To test whether these developmental phenotypes were, as for the AMP induction, consecutive to IMD pathway activation, we performed epistasis experiments. Inactivation of *IMD*, *Dredd*, *Kenny*, *Relish* but not the JNK mediator *Hemipterous*, completely suppressed abdominal tergites and genitalia orientation defects of *PGRP-LF* mutants (Fig 5A and S6A Fig). Similar results were obtained when PGRP-LF was inactivated only in its expression domain via RNA interference (Fig 5A). This suggests that uncontrolled activation of IMD pathway in cells that are fated to generate these structures is incompatible with proper developmental processes. We further tested this hypothesis by analyzing the consequences associated with a constitutive IMD pathway activation in cells that will give rise to tergites and genitalia. Ectopic expression of either IMD or PGRP-LC, whose overexpression is sufficient to activate IMD signaling in the absence of bacteria, in the PGRP-LF expression domain fully phenocopied *PGRP-LF* mutant in tergites and genitalia (S7A Fig). Only one of the two adult phenotype was observed when Gal4 drivers specific to either tergites (*Ddc*^*Gal4*^) or genitalia (*AbdB*^*LDN-Gal4*^) was used (S7A Fig). These lines specifically target larval epidermal cells (*Ddc*^*Gal4*^) and the outer ring of cells of the A8 abdominal segment (*AbdB*^*LDN-Gal4*^), both being cells in which induction of apoptosis is essential for proper development. Altogether, these results demonstrate that PGRP-LF function is needed, in the absence of bacterial infection, to prevent constitutive activation of IMD pathway in ectoderm, which will otherwise provoke developmental defects.

### PGRP-LF inactivation and IMD activation is blocking apoptosis

**Fig 6:**
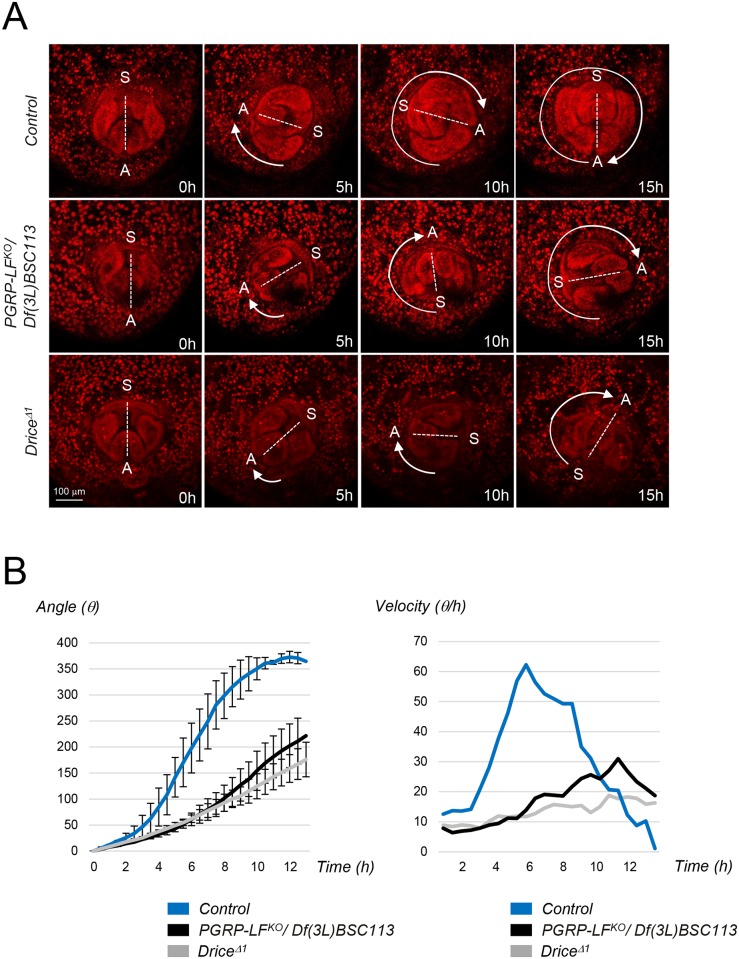
Genitalia rotation is impaired in *PGRP-LF* mutants. (A) Time-lapse series of genitalia rotation in *His2Av-mRFP/+* (control), *His2Av-mRFP/+; PGRP-LF*^*KO*^*/Df(3L)BSC113* and *His2Av-mRFP/+; Drice*^*Δ1*^/*Drice*^*Δ1*^ pupae. Ventral is towards the top in all panels. A and S indicate respectively anus and sexe primordia locations. (B) The genitalia angle (θ) in control (blue), *PGRP-LF*^*KO*^*/Df(3L)BSC113* (black) and *Drice*^*Δ1*^mutant pupae (gray) was measured every 30 minutes, and the mean angle is shown. Error bars indicate SD (control, *n* = 5; *PGRP-LF*^*KO*^, *n* = 6, *Drice*^*Δ1*^ *n* = 5). Velocity (*V* = *d*θ */dt*) was quantified by measuring θ as a function of time *t* in control (blue), *PGRP-LF*^*KO*^*/Df(3L)BSC113* (black) and *Drice*^*Δ1*^ mutant (gray).

During metamorphosis, tergites and male genitalia development requires apoptosis [41–45]. Consistently, mutations in apoptotic genes such as *Drice*, or overexpression of the apoptosis inhibitors P35 or Diap1 in tergite and genitalia anlage, give rise to adult flies presenting cuticle midline and genitalia rotation defects [41]. To test if *PGRP-LF* mutant phenotypes are due to apoptosis inhibition in ectodermal anlage, we overexpressed P35 in PGRP-LF expression domain. *PGRP-LF*^*Gal4*^, *UAS-P35* flies fully phenocopied *PGRP-LF* mutants. Although, such phenotypes were not observed with a weaker *PGRP-LF*^*Gal4*^ driver, it occurred in flies heterozygous for the *PGRP-LF*^*KO*^ allele (S7B Fig). To further confirm these results, we compared the morphogenetic movements associated with the formation of these ectodermal structures in wild-type and *PGRP-LF* mutants. First, we investigated tergite patterning which requires replacement of larval abdominal epidermis with adult epithelium during pupariation. During this process, larval epidermal cells (LECs) undergo caspase-dependent apoptosis and are progressively replaced by histoblasts that expand as the LECs die. Inhibition of apoptosis in LECs by P35 overexpression or by *Drice* inactivation (Fig 5B), leads to the persistence and accumulation of LECs along the dorsal midline of the pupal epidermis. A very similar LECs accumulation was observed in *PGRP-LF* mutant pupae but not in controls (Fig 5B). To monitor apoptosis in these tissues, we took advantage of the anti-Dcp1 antibody that specifically recognizes the cleaved from of the effector caspase and therefore labels dying cells. Using such a tool, we detected a reduced number of LECs undergoing apoptosis in *PGRP-LF* mutant pupae, when compared to controls (Fig 5C and 5D). The fact that PGRP-LF is expressed in LECs but absent from the histoblasts, suggests that it is required tissue autonomously in LECs to allow proper induction of apoptosis (S7D Fig). We next examined male genitalia rotation using video recording of controls and *PGRP-LF* mutant pupae. During pupariation, the male genitalia rotates 360° clockwise and the acceleration and full completion of this process requires apoptosis of specific cells located in the *AbdB* expressing segment. We found that if genitalia of control flies rotate of 360° within 15 hours, those of *PGRP-LF* mutants only reach a 240° rotation during this period (Fig 6A). Moreover, both the angle and the velocity of the genitalia rotation were reduced in *PGRP-LF* mutants compared to wild-type although they were not as affected as in a *Drice*^Δ1^ mutants (Fig 6A and 6B). Hence, genetic data and live imaging data demonstrate that the lack of PGRP-LF protein affects developmental processes by preventing normal apoptosis to occur in these tissues.

### IMD pathway activation blocks apoptosis by modulating Diap1 expression

**Fig 7:**
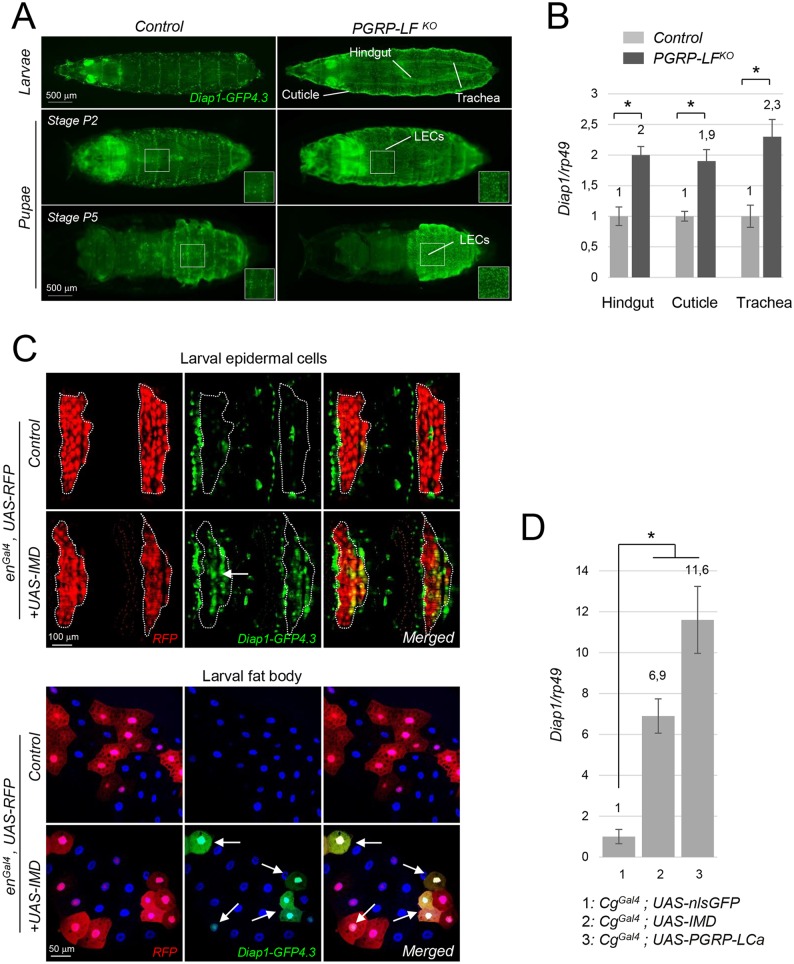
Activation of Diap1 expression in *PGRP-LF* mutants and IMD gain-of-function cells. (A) Dorsal views of third instar larvae and pupal cases showing ectopic expression of *Diap1-GFP4*.*3* in *PGRP-LF* mutant LECs, trachea and hindguts. See also S7 Fig. (B) Expression of *Diap1* mRNA is higher in hindgut, trachea and cuticle from *PGRP-LF* mutant larvae than from controls. (C) *Diap1-GFP4*.*3* expression is induced in cells overexpressing IMD. Dorsal epidermis (top images) and fat body (bottom images) of *en*^*Gal4*^, *UAS-RFP*/+ (control) and *en*^*Gal4*^, *UAS-RFP*/+; *UAS-IMD*/+ (*UAS-IMD*) larvae are shown. In LECs and fat body cells, IMD expression induce *Diap1-GFP4*.*3* cell autonomously (arrows). (D) Expression of *Diap1* mRNA is induced following overexpression of *IMD* and *PGRP-LCa* in adult fat body. Abdomen from 6d old females of the following genotypes were dissected and analyzed by q-RT-PCR: *Cg*^*Gal4*^*/nlsUAS-GFP* (control), *Cg*^*Gal4*^/+*; UAS-IMD*/+ (*UAS-IMD*) and *Cg*^*Gal4*^/+*; UAS-PGRP-LCa*/+ (UAS-PGRP-LCa). For (B) and (D) mRNA level in control was set to 1, and values obtained with tissues or larvae of indicated genotypes were expressed as a fold of this value. For (B) and (D) histograms correspond to the mean value ± SD of three independent experiments. Values indicated by symbols (*) are statistically significant (t-test, p < 0.05). ns: not significantly different.

IMD pathway could block apoptosis by either repressing pro-apoptotic genes or by activating apoptosis inhibitors [46]. To distinguish between these possibilities, we compared expression levels of pro and anti-apoptotic genes in *PGRP-LF* mutants and in controls. First, using the *Diap1-GFP4*.*3* and the *PGRP-LF*^*Gal4*^ transgenes, we analyzed the expression pattern of *Diap1* and *PGRP-LF* in wild type conditions. In both L3 and pupae, *PGRP-LF*^*Gal4*^ was expressed in all LECs expected for one row of cells repeated along the antero-posterior axis (S8A Fig). Remarkably, although *Diap1-GFP4*.*3* expression was weak in most LECs, it was strongly expressed in these cells that do not express PGRP-LF, suggesting that PGRP-LF could repress *Diap1-GFP4*.*3* expression. We next compared *Diap1-GFP4*.*3* expression in wild-type and *PGRP-LF* mutant larvae and pupae. PGRP-LF inactivation was associated with an ectopic expression of *Diap1-GFP4*.*3* in all LECs that became uniformly positive for *Diap1-GFP4*.*3*, but also in other PGRP-LF expressing tissues such as the trachea and the hindgut (Fig 7A and S9A and S9B Fig). q-RT-PCR quantification confirmed the increased expression of *Diap1* mRNAs in *PGRP-LF* mutant larval tissues such as the cuticle, the hindgut and the trachea, when compared to controls (Fig 7B). Consistently, ectopic activation of the IMD pathway in the larval epidermis and in the fat body was sufficient to induce ectopic expression of *Diap1-GFP4*.*3* transgene in an autonomous fashion (Fig 7C) and this was confirmed by q-RT-PCR quantification (Fig 7D). In contrast, neither loss-of function of *PGRP-LF* nor gain-of-function of *IMD* were able to activate pro-apoptotic *Hid* or *Reaper* transgenic reporter constructs in these tissues (S10A–S10C Fig). We could also showed that Diap1 ectopic expression using either a ubiquitous Gal4 (*Act5C*^*Gal4*^) or *PGRP-LF*^*Gal4*^ drivers was sufficient to mimic both cuticle and genitalia defects (S7C Fig) [41]. Altogether these results show that by preventing IMD pathway activation in ectodermal derivatives, PGRP-LF is allowing normal cell death to occur and pupal development to proceed.

## Discussion

We showed here that one of essential role of PGRP-LF is to prevent a bacteria independent constitutive activation of the NF-κB pathway, which otherwise perturbs tergite and genitalia formation during pupariation. We also confirmed previous data showing that a lack of IMD pathway repression by PGRP-LF is leading to AMP production in ectodermal derivatives [33]. Both *PGRP-LF* expression pattern and loss-of-function phenotype analyses showed that PGRP-LF is mainly acting in ectodermal cells. This is mostly evident in the intestinal tract that is formed during embryogenesis by associating domains of both mesodermal and ectodermal origins. Although IMD pathway is essential in regulating antibacterial response in the mesodermal derived midgut, PGRP-LF is only playing a minor role as an IMD regulator in this tissue. This contrasts with its importance in both neighboring ectodermal derivatives that are the fore and the hindgut. Loss of PGRP-LF function triggers in these tissues a massive AMP production. Interestingly also, is the fact that the effects of inactivating PGRP-LF, and hence of IMD pathway permanent activation, are not the same in all ectodermal derivatives. Whereas in trachea, epidermis or hind/foregut, it only leads to AMP constitutive production, it has profound and deleterious effects on tergites and genitalia. Of note, these are the two known structures whose proper morphogenesis has been shown via P35 overexpression to depend on caspase activity [41]. Removing PGRP-LF, blocks apoptosis and in turn interferes with developmental processes of these adult structures. It could be that apoptosis is also prevented in other ectodermal derivatives but that this has no impact on their development. Since mutations in caspases cause pleiotropic defects during development, it is obvious that PGRP-LF is only antagonizing a limited fraction of them, consistently with its restricted spatial pattern [47].

The fact that IMD pathway constitutive activation is blocking apoptosis is unexpected since previous results rather spoke for a pro-apoptotic function of IMD activation [48, 49]. Ectopic activation of the IMD protein was shown to induce apoptosis in fat body cells while ectopic expression of the anti-apoptotic protein P35 was shown to prevent IMD induced AMP production [48]. In our hands, ectopic activation of IMD in imaginal discs, generates visible IMD pathway dependent phenotypes that were not suppressed by P35 overexpression (S11 Fig and S1 Table). These results suggest that IMD pathway activation can have very different consequences depending on the cellular context. The rationale behind this could reflect the properties of the infected tissues. In proliferating epithelial tissues, such as the midgut or imaginal discs, dying cells induce their neighbors to divide to compensate for the lost space [50, 51]. In such tissues, IMD pathway mediated cell death following infection should be without deleterious consequences. However, in cells that have left the mitotic cycle such compensatory proliferation cannot be a tissue repair system. Recent work has shown that these tissues are instead relying on compensatory cell hypertrophy and polyploidization for wound healing [52, 53]. One can imagine that IMD triggered cell death in such tissues should be as much as possible prevented. PGRP-LF could play such a role.

The results of this study reinforced the idea that in the absence of PGRP-LF, IMD pathway is constitutively active in the complete absence of bacterial product. This raises the question of the mechanisms of PGRP-LC mediated IMD activation. One possibility is that an endogenous ligand not derived from bacteria and probably endogenous is able to activate the IMD pathway. Before this putative elicitor is identified, this hypothesis would be difficult to prove. However, since PGRP-LC cleavage (as for other receptor such as Notch) has been shown to be sufficient for its activation and hence for IMD pathway triggering, endogenous proteases are good candidate for such PGRP-LC bacteria independent activators [54, 55]. Consistently, clean wounding of the epidermis is sufficient to trigger AMP production in the absence of any bacteria [56]. Since embryonic development, and specially metamorphosis during which tergites and genitalia form, rely on extensive tissue remodeling including protease expression and release to eliminate dead tissues, this hypothesis is worth considering. Alternatively, PGRP-LC activation could take place in the complete absence of ligand. Crystal structure experiments have shown that the presence of PGRP-LF at the cell surface is reducing the probability of forming unwanted signaling PGRP-LC dimers in the absence of bacteria ligand hence contributing to the maintenance of a low IMD background level [34]. In this case, AMP expression in PGRP-LF deficient ectodermal tissues will be due to a PGRP-LC spontaneous dimerization sufficient to trigger signalling. Finally, PGRP-LF has been shown to compete with PGN derived ligand TCT for binding to PGRP-LC. By doing so, PGRP-LF could also increase the thresholds at which IMD pathway is activated by the presence of bacteria derived elicitor. Cells expressing PGRP-LF will require more PGN to activate the IMD pathway than cells devoid of it. It is interesting to note that PGRP-LF is expressed in foregut and hindgut epithelia that are not protected by a peritrophic membrane and are therefore in direct contact with bacteria. The presence of PGRP-LF at the membrane of such cells would insure that IMD pathway activation is only acting when a certain concentration of PGN elicitor is reached. Very transient and weak PGN level fluctuations will not be detected in these regions preventing responses to spurious stochastic events in the enterocytes. In contrast, neighboring midgut enterocytes are separated from bacteria by the peritrophic membrane that only allows diffusion of molecules such as nutriments or bacteria derived molecules. In the case of high bacteria loads, it is expected that high amount of PGN are released by bacteria, cross the peritrophic membrane and reach the midgut enterocytes. Detection of PGN by midgut enterocytes would therefore be the sign of a gut infection and should be followed by IMD pathway activation. The presence of PGRP-LF in such cells will prevent IMD activation. The same idea will hold true for the fat body cells in which PGRP-LF is playing a minor role. Fat body cells are sampling the circulating hemolymph to detect the presence of PGN that, in uninfected flies, should not be present in the body cavity. This sensing mechanism should be as sensitive as possible and not buffered by the presence of PGRP-LF at the fat body cell membranes. We propose here that the presence of PGRP-LF as an IMD pathway activation modulator is depending of cell types and on their role in the antibacterial response.

The *PGRP-LF*^*KO*^ mutant that we have generated is sublethal with few escapers showing tergites and genitalia defects but normal wings. We have demonstrated that these developmental defects are likely due to an apoptosis blockage. The previously characterized *PGRP-LF*^*200*^ *allele* did not present cuticle and genitalia defects but had wing notchings that were also observed by reducing PGRP-LF levels via RNAi in wing imaginal discs. These defects were associated with ectopic activation of the JNK pathway and increased apoptosis. To try to explain these discrepancies, we have characterized the *PGRP-LF*^*200*^ allele that was obtained by P element mobilization. Our results show that if the *PGRP-LF*^*200*^ *allele* has lost the white cDNA that serves as an eye marker for the P-element, it has kept some of the P element sequence 5’ to PGRP-LF coding region that might interfere with neighboring gene expression (S1 Fig). Q-RT-PCR analysis indicates that PGRP-LF mRNA levels are globally not affected in these flies that do also not show ectopic *Diptericin* transcription. We hypothesized that wing notching in *PGRP-LF*^*200*^ flies are more likely due to an imaginal disc specific modulation of the relative PGRP-LF/PGRP-LC ratio than to a global loss of PGRP-LF function. Interestingly, a recent study identified some of the NF-κB pathway components, among which PGRP-LC, as proteins involved in a surveillance of cell fitness and cell competition in wing imaginal discs [57]. This might explain why elimination of PGRP-LF function in the entire wing imaginal disc (such as in *PGRP-LF*^*KO*^) or some domains only (RNAi to PGRP-LF) give rise to different phenotypes.

## Materials and Methods

### *Drosophila melanogaster* strains and maintenance

The following strains were used in this work: PGRP-LF^Gal4weak^ (this work), PGRP-LF^Gal4strong^ (this work), PGRP-LC^Gal4^ (this work), UAS-nlsGFP BL#4775, UAS-myr-mRFP BL#7118, Ddc^Gal4^ BL#7010, AbdB^LDNGal4^ BL#55848, Fng^Gal4^ BL#9891, ap^md544Gal4^ BL#3041, en^Gal4^, UAS-RFP BL#30557, Act5C^Gal4^ BL#25374, UAS-mcd8CherryRFP BL#27392; AttacinD-Cherry (this work), Diptericin-Cherry [58], Drosomycin-GFP [59], Diap1-GFP4.3 [60], hid5’F-GFP, rprNP0520^Gal4^ DGRC#103634, His2Av-mRFP BL#23651, imd^shadok^ [48], PGRP-LE^112^ [8], PGRP-LC^ΔE12^ [61], dMyd^88c03881^ [62], Dredd^D55^ [63], Diap^2c7c^ [64], Relish^E20^ [65], pirk^EY0073^ [31], PGRP-LB^KO^ [40], dTak1 [66], Dcp-1^Prev1^BL#63814, Drice^Δ1^ [67], UAS-P35 [48], UAS-PGRP-LCa BL#30917, UAS-p53 BL#8418, UAS-Diap1 BL#63820, UAS-PGRP-LF-IR (this work), UAS-Dicer2 BL#24650. Flies were grown at 25°C on a yeast/cornmeal medium. For 1l of food, 8.2g of agar (VWR, cat. #20768.361), 80g of cornmeal flour (Westhove, Farigel maize H1) and 80g of yeast extract (VWR, cat. #24979.413) were cooked for 10 min in boiling water; 5.2 g of Methylparaben sodium salt (MERCK, cat.#106756) and 4 ml of 99% propionic acid (CARLOERBA, cat. #409553) was added when the food had cooled down. For antibiotic treatment, standard medium was supplemented with Ampicillin, Kanamycin, Tetracyclin and Erythromycin at 50 μg/ml final concentrations.

### Mutant generation

*PGRP-LF*^*KO*^ line was generated by homologous recombination. The PGRP-LF gene was replaced by a mini-white gene. DNA flanking the 5’ and 3’ ends used were respectively, 2,914 bp and 3,060 bp for PGRP-LF locus. Sequences were cloned in the pW25 vector [68].

### Imaging

Larval, pupal or adult tissues were dissected in PBS, fixed for 20 min in 4% paraformaldehyde on ice and rinse 3 times in PBT (PBS + 0.1% Triton X-100). For antibody staining on pupal cases, cleaved anti-Dcp-1 antibody (Cell Signaling #9578) was used at 1:200. The tissues were mounted in Vectashield (Vector Laboratories) fluorescent mounting medium, with or without DAPI. Images were captured with either a Stereo Discovery V12 microscope or a LSM 780 Zeiss confocal microscope.

### Sample preparation for time-lapse imaging microscopy

Staged pupae (24 hours APF) were washed in water and mounted on a glass slide using a drop of silicon grease (Dow corning). The pupal case covering the caudal part of the abdomen was removed. A very wet filter paper was placed around the pupae to keep them hydrated. The pupae were covered with a cover glass in a small drop of water to avoid desiccation. High-vacuum silicone grease (Dow Corning) was also used to seal the chamber. In most cases, the animal survived the data acquisition and developed into an adult. Time-lapse images were captured using a Nikon Macroconfocal AZ100.

### Quantitative real-time PCR

RNA from whole larvae or dissected organs (n = 30) was extracted with RNeasy Mini Kit (QIAGEN, cat. #74106). Quantitative real-time PCR, TaqMan, and SYBR Green analysis were performed as previously described [36]. Primers information can be obtained upon request. The amount of mRNA detected was normalized to control rp49 mRNA values. Normalized data was used to quantify the relative levels of a given mRNA according to cycling threshold analysis (ΔCt).

### Infection of adults by Ecc^15^

The bacterial strain used was *Erwinia carotovora carotovora 15 2141* (Ecc) cultured in Luria-Bertani medium at 30°C overnight. Bacterial cultures were centrifuged at 2500 g for 15 min at RT and resuspended in fresh Luria-Bertani medium. Cells were serially diluted in PBS and their concentration was determined by optical density (OD) measurement at 600 nm. For oral infection, flies were first incubated 2 hr at 29°C in empty vials and then placed in a fly vial with food. The food solution was obtained by mixing a pellet of an overnight culture of bacteria Ecc-15 (OD = 200) with a solution of 5% sucrose (50/50) and added to a filter disk that completely covered the agar surface of the fly vial. Septic injuries were performed by pricking adult females with a thin needle contaminated with *Ecc-15*.

### Survival tests with bacterial infection

For oral infections, adult flies were infected every 2 days with a solution of *Ecc* (OD = 200) 5% sucrose (50/50). For septic injuries, adult females were pricked once with a thin needle contaminated with *Ecc*. At least two tubes of 20 flies were used for each survival assay and three replicates of this experiment were done. Survival was scored several times a day.

## Supporting Information

S1 Fig Generation and characterization of *PGRP-LF*^*KO*^ mutant allele. (A) Schematic representation of the *PGRP-LF* locus and of the *PGRP-LF*^*200*^ and *PGRP-LF*^*KO*^ mutants. The gene map was adapted from FlyBase. The deleted segment in *PGRP-LF*^*KO*^ that was replaced by the mini-white gene is indicated. The deletion in *PGRP-LF*^*KO*^ starts at position 3L: 9,350,539 and ends at position 3L: 9,349,489. The *PGRP-LF*^*200*^ mutant was obtained by P-element mobilisation and screening for the loss of the white eye marker [33]. Sequencing reveals that a piece of P-element transposon deleted of the white cDNA is still present in *PGRP-LF*^*200*^ mutants (B) Relative gene expression of *PGRP-LF*, *PGRP-LC and Diptericin mRNA* in *PGRP-LF*^*200*^ and *PGRP-LF*^*KO*^ mutants compared to controls. For (B), histograms correspond to the mean value ± SD of three independent experiments. Values indicated by symbols (*) are statistically significant (t-test, p < 0.05). ns: not significantly different.
(TIF)

S2 Fig PGRP-LF and PGRP-LC are expressed in ectodermal derivatives. (A) *PGRP-LF*^*Gal4strong*^, *UAS-nlsGFP* and *PGRP-LF*^*Gal4weak*^, *UAS-nlsGFP* larvae showing GFP expression in salivary glands, foregut, hindgut and cuticle and to a lesser extend in fat body. (B) mRNA enrichment values from Rp49, PGRP-LF and PGRP-LC in third instar larvae tissues. Data are from FlyAtlas. (C) A) *PGRP-LC*^*Gal4*^, *UAS-nlsGFP* larvae showing GFP expression in trachea, foregut, hindgut and cuticle.
(TIF)

S3 Fig *PGRP-LF*^*KO*^ *molecular* characterization. *(A)* Relative gene expression of *PGRP-LC*, *UGP and PGRP-LF in PGRP-LF*^*KO*^. (B) *PGRP-LC* mRNA expression in flies mutants for *PGRP-LF* and *IMD* pathway components. Histograms correspond to the mean value ± SD of three independent experiments. Values indicated by symbols (*) are statistically significant (t-test, p < 0.05). ns: not significantly different.
(TIF)

S4 Fig PGRP-LF expression in ectodermal derivatives prevents constitutive AMP production. (A) Overexpression of AMP reporter genes in *PGRP-LF* mutant larvae. Ectopic *Diptericin-Cherry* and *Drosomycin-GFP* expression are detected in the trachea and cuticle (*Dipt-Cherry* only) of *PGRP-LF* mutant larvae (either *PGRP-LF*^*KO*^ or *PGRP-LF*^*KO*^*/Df(3L)BSC113*). (B) Ectopic expression of *AttacinD-Cherry* is detected in *PGRP-LF*^*Gal4*^*; UAS-nlsGFP* expressing tissues of *PGRP-LF*^*KO*^ mutant larvae. (C) Overexpression of *Drosomycin-GFP* expression in the trachea is suppressed in *Dredd*^*D55*^*; PGRP-LF*^*KO*^ double mutant larvae.
(TIF)

S5 Fig *Diptericin* expression in orally infected *PGRP-LF*^*RNAi*^ flies. Fat body IMD pathway activation, monitored by *Diptericin* expression, 24h after *Ecc* oral infection. PGRP-LF inactivation by RNAi (*PGRP-LF*^*Gal4strong*^*; UAS-PGRP-LF*^*RNAi*^*/UAS-Dicer2*) modifies IMD pathway inducibility in the fat body of *Ecc* orally infected flies as observed following complete inactivation of PGRP-LF in *PGRP-LF*^*KO*^*/Df(3L)BSC113* flies. mRNA level in controls was set to 1, and values obtained with indicated genotypes were expressed as a fold of this value. Histograms correspond to the mean value ± SD of three independent experiments. Values indicated by symbols (*) are statistically significant (t-test, p < 0.05). ns: not significantly different.
(TIF)

S6 Fig Male genitalia anatomy is not affected in *PGRP-LF* mutants. (A) Magnification of the pictures shown in Fig 5A. A and S indicate respectively anus and sexe primordia locations. (B) The external anatomy of both the male and female genitalia are not affected by the PGRP-LF mutation.
(TIF)

S7 Fig Inactivation of *PGRP-LF* by RNAi or IMD pathway overactivation induce abdominal cuticle and male genitalia defects. (A-C) Dorsal (d) and ventral (v) views of male or female adult abdomen showing incomplete epidermis differentiation (white arrow) and abnormal male genitalia orientation (white bar). (A) 1, 2: Overexpression of *IMD* in LECs with *Ddc-Gal4* prevents normal dorsal epidermis fusion (arrow). Abnormal male genitalia orientation in flies overexpressing (3) *P35*, (4) *IMD* or (5) *PGRP-LCa* in AbdB expressing cells (white bar). Abnormal genitalia rotation and incomplete epidermis differentiation are both observed in *PGRP-LF*^*Gal4strong*^ */ UAS-IMD* or *PGRP-LF*^*Gal4strong*^ */ UAS-PGRP-LC*a flies. (B) Abdomen from adults overexpressing *P35* under the control of (1) *PGRP-LF*^*Gal4strong*^ or (2 and 3) *PGRP-LF*^*Gal4weak*^ drivers. Incomplete fusion of dorsal epidermis and abnormal genitalia rotation are observed with *PGRP-LF*^*Gal4strong*^ or with *PGRP-LF*^*Gal4weak*^ when flies lack one copy of *PGRP-LF* gene. (C) Abdomen from adults overexpressing *Diap1* under the control of (1 and 2) *PGRP-LF*^*Gal4strong*^ or (3 and 4) *Act5C*^*Gal4*^ drivers. (D) Lateral views of *PGRP-LF*^*KO*^ pupal cases expressing *PGRP-LF*^*Gal4*^*; UAS-nlsGFP* and *AttacinD-Cherry* 24h APF. *PGRP-LF*^*Gal4*^ and *AttacinD-Cherry* expression are both restricted to LECs and absent from histoblasts. The dashed lines indicate the boundary between the histoblasts and LECs.
(TIF)

S8 Fig *PGRP-LF* and *PGRP-LC* expression in third instar larval and pupal epidermal cells. Dorsal view of *Diap1-GFP4*.*3*; *PGRP-LF*^*Gal4*^, *UAS-mcd8CherryRFP* (A) or *Diap1-GFP4*.*3*; *PGRP-LC*^*Gal4*^, *UAS-mcd8-CherryRFP* (B) third instar larvae or pupae 24h APF. (A) *PGRP-LF*^*Gal4*^ is expressed in almost all dorsal LECs except in cells expressing strongly *Diap1-GFP4*.*3* (arrows in A). (B) *PGRP-LC*^*Gal4*^ is expressed in some dorsal LECs and is co-expressed with cells expressing strongly *Diap1-GFP4*.*3* (arrows in B).
(TIF)

S9 Fig *Diap1-GFP4*.*3* is induced in larval tissue mutant for *PGRP-LF*. (A) Confocal images of dissected larval trachea and hindgut showing enhanced expression of *Diap1-GFP4*.*3* in *PGRP-LF* mutant tissue when compared to controls. The dashed lines indicate the periphery of the control tissues. (B) *Diap1-GFP4*.*3* is ectopically expressed in ectodermal derivatives of the larval gut of *PGRP-LF* mutants. Both the foregut (arrow) and the hindgut enterocytes (arrow head) express *Diap1-GFP4*.*3* in *PGRP-LF* mutants compare to controls.
(TIF)

S10 Fig *Hid* or *Reaper* reporter genes are not induced in *PGRP-LF* mutants or in IMD gain of function mutant cells. *Hid-GFP* expression is not induced in cells overexpressing IMD. Dorsal epidermis (A) and fat body (B) of *en*^*Gal4*^, *UAS-RFP*/+ (control) or *en*^*Gal4*^, *UAS-RFP*/+; *UAS-IMD*/+ (*UAS-IMD*) larvae are shown. In both LECs and fat body cells, expressing IMD do not induce expression of *Diap1-GFP4*.*3* while in LECs *p53* overexpression does (arrows). (C) *Reaper*^*Gal4*^*; UAS-nlsGFP* expression is not ectopically expressed in LECs from third instar *PGRP-LF* mutant larvae.
(TIF)

S11 Fig Effects of IMD pathway ectopic expression in wings. Adult wings from 5d old males of indicated genotype. Overexpression of *IMD* in the dorsal part of the wing pouch using *ap*^*Gal4*^ driver induces wing bubbles, a phenotype that is not suppressed by co-expression of the anti-apoptotic protein P35.
(TIF)

S1 Table Genetic interactions between IMD and apoptotic signaling pathways. Genetic interactions showing that lethality due to IMD gain-of-function is not suppressed, but rather enhanced, when apoptosis is blocked.
(TIF)
