# Supplementary figures and images for: Inhibition of a NF-κB/Diap1 Pathway by PGRP-LF Is Required for Proper Apoptosis during Drosophila Development
Source: PLoS Genet. 2017 Jan 13;13(1):e1006569. doi: 10.1371/journal.pgen.1006569 (PMC5279808; doi:10.1371/journal.pgen.1006569)

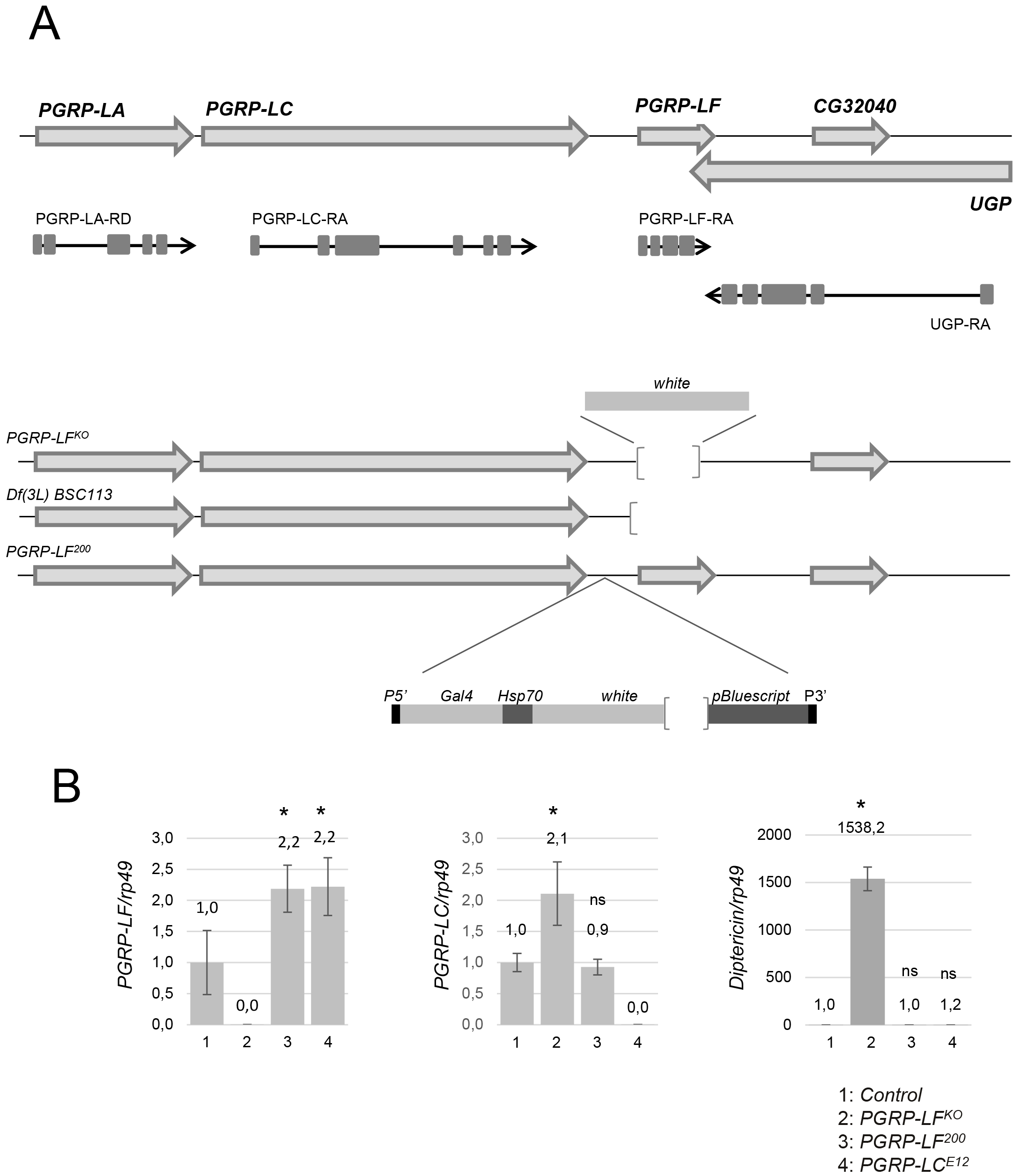

Supplement: S1 Fig — (A) Schematic representation of the PGRP-LF locus and of the PGRP-LF200 and PGRP-LFKO mutants. The gene map was adapted from FlyBase. The deleted segment in PGRP-LFKO that was replaced by the mini-white gene is indicated. The deletion in PGRP-LFKO starts at position 3L: 9,350,539 and ends at position 3L: 9,349,489. The PGRP-LF200 mutant was obtained by P-element mobilisation and screening for the loss of the white eye marker [33]. Sequencing reveals that a piece of P-element transposon deleted of the white cDNA is still present in PGRP-LF200 mutants (B) Relative gene expression of PGRP-LF, PGRP-LC and Diptericin mRNA in PGRP-LF200 and PGRP-LFKO mutants compared to controls. For (B), histograms correspond to the mean value ± SD of three independent experiments. Values indicated by symbols (*) are statistically significant (t-test, p < 0.05). ns: not significantly different. (TIF) [file pgen.1006569.s001.tif]

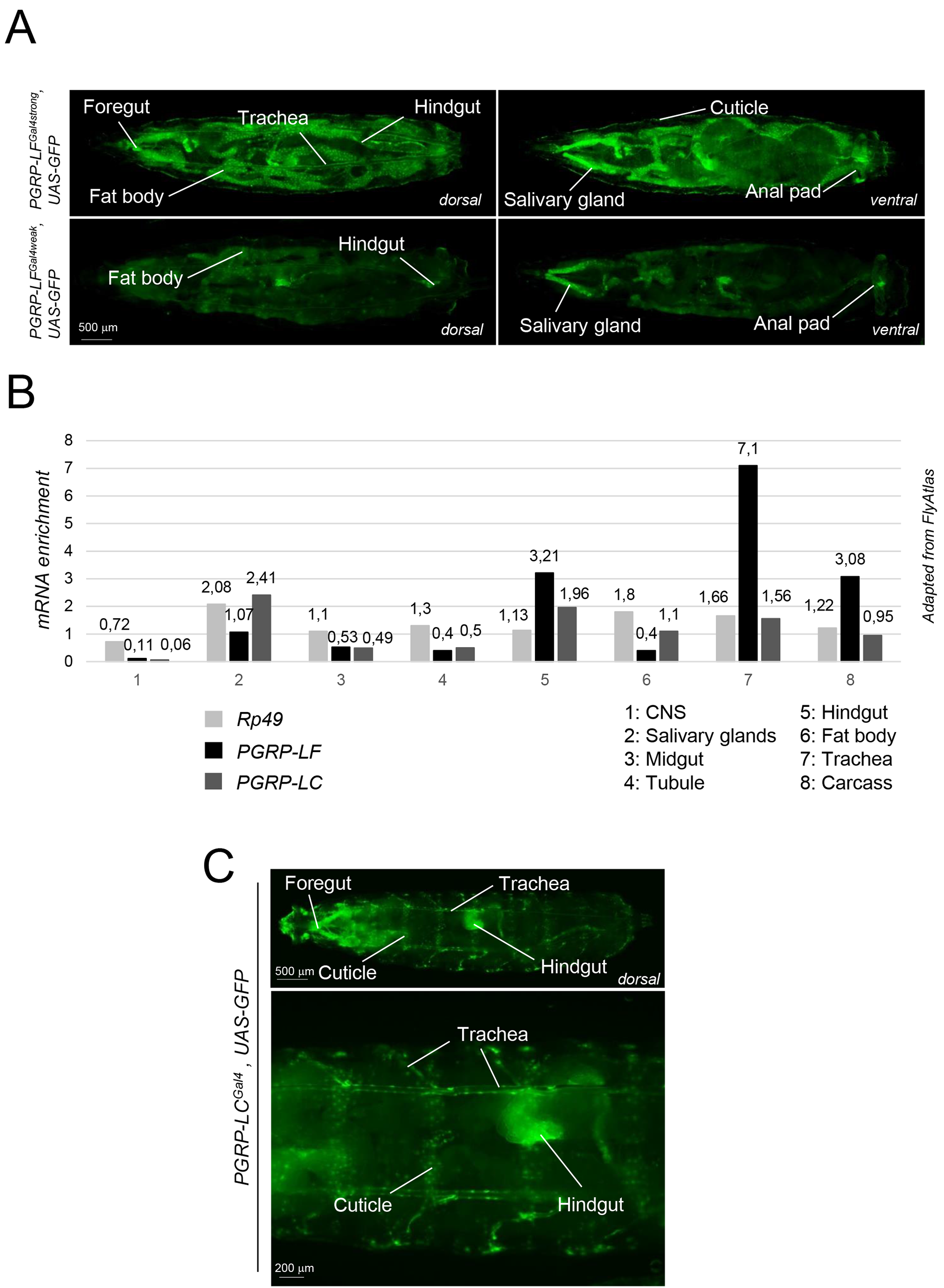

Supplement: S2 Fig — (A) PGRP-LFGal4strong, UAS-nlsGFP and PGRP-LFGal4weak, UAS-nlsGFP larvae showing GFP expression in salivary glands, foregut, hindgut and cuticle and to a lesser extend in fat body. (B) mRNA enrichment values from Rp49, PGRP-LF and PGRP-LC in third instar larvae tissues. Data are from FlyAtlas. (C) A) PGRP-LCGal4, UAS-nlsGFP larvae showing GFP expression in trachea, foregut, hindgut and cuticle. (TIF) [file pgen.1006569.s002.tif]

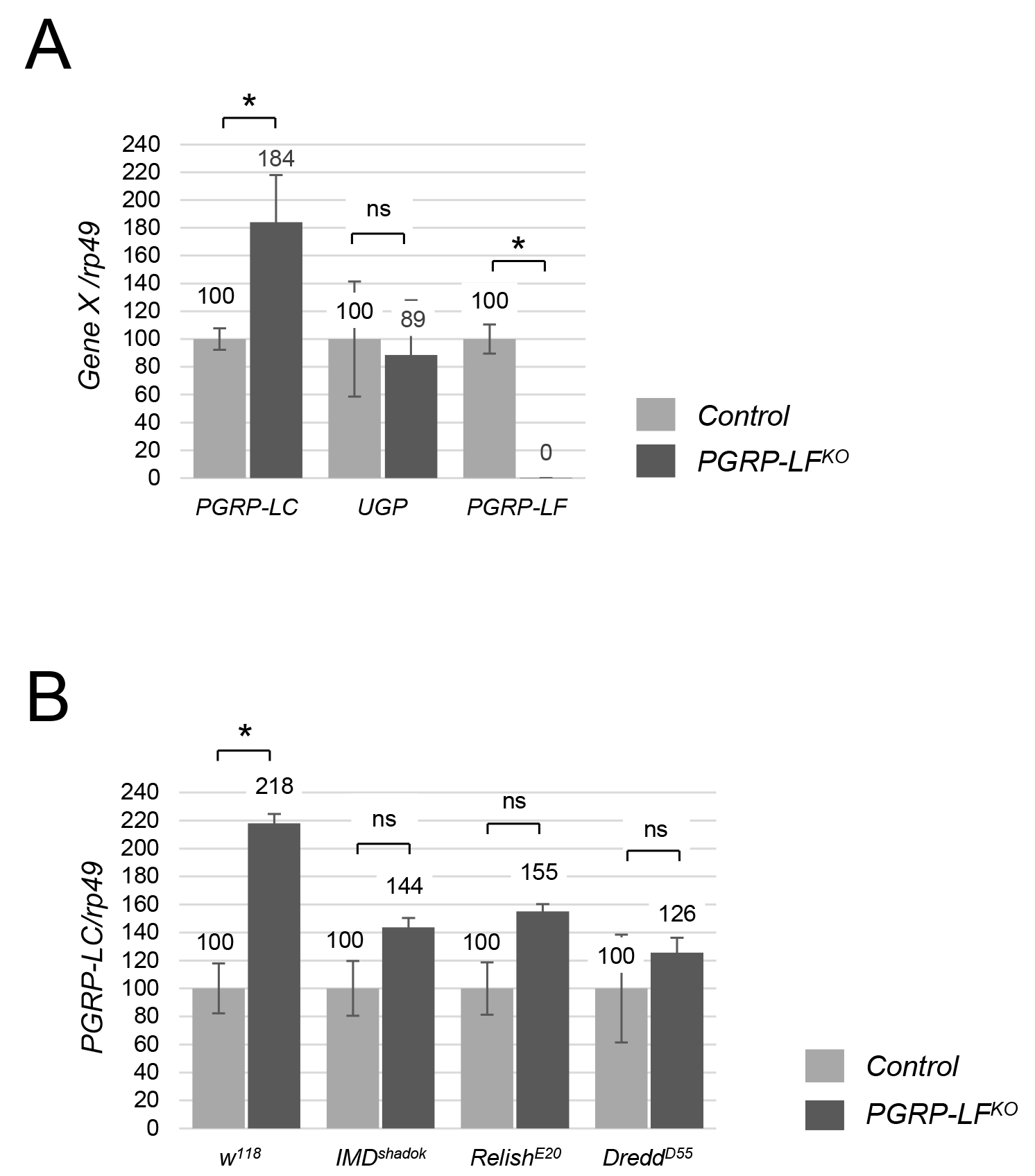

Supplement: S3 Fig — (A) Relative gene expression of PGRP-LC, UGP and PGRP-LF in PGRP-LFKO. (B) PGRP-LC mRNA expression in flies mutants for PGRP-LF and IMD pathway components. Histograms correspond to the mean value ± SD of three independent experiments. Values indicated by symbols (*) are statistically significant (t-test, p < 0.05). ns: not significantly different. (TIF) [file pgen.1006569.s003.tif]

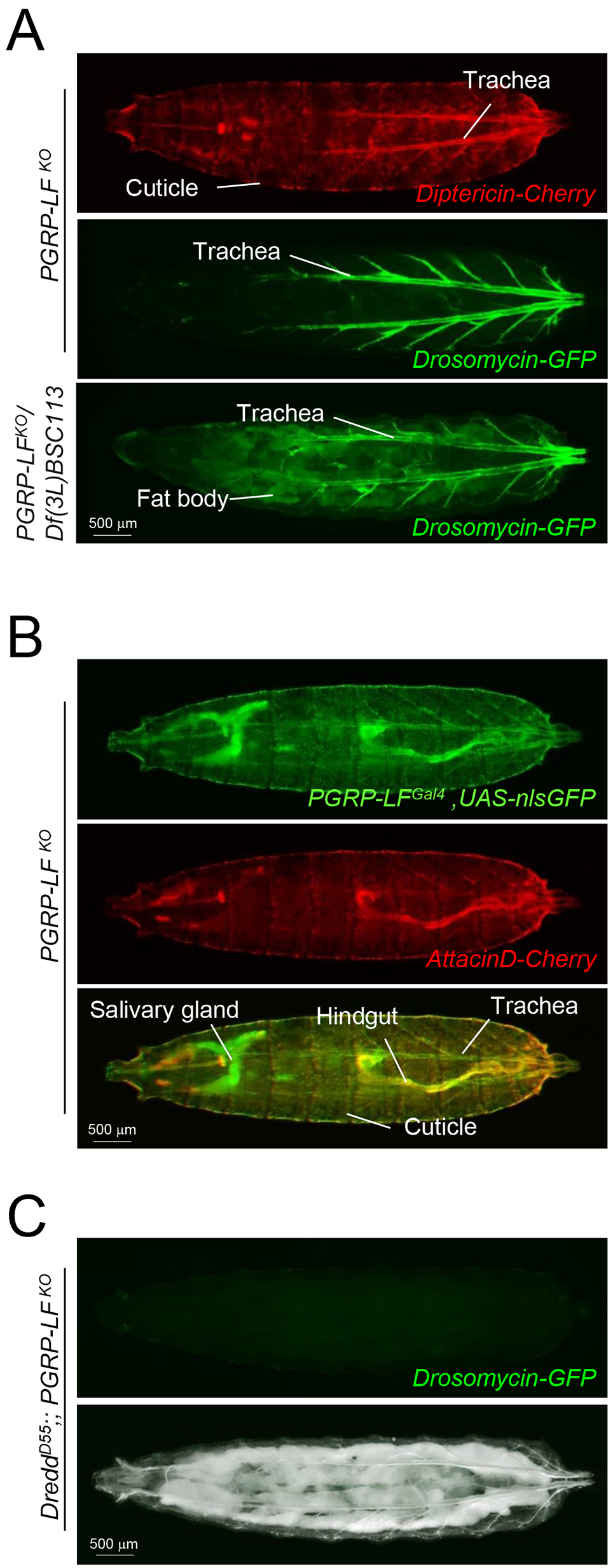

Supplement: S4 Fig — (A) Overexpression of AMP reporter genes in PGRP-LF mutant larvae. Ectopic Diptericin-Cherry and Drosomycin-GFP expression are detected in the trachea and cuticle (Dipt-Cherry only) of PGRP-LF mutant larvae (either PGRP-LFKO or PGRP-LFKO/Df(3L)BSC113). (B) Ectopic expression of AttacinD-Cherry is detected in PGRP-LFGal4; UAS-nlsGFP expressing tissues of PGRP-LFKO mutant larvae. (C) Overexpression of Drosomycin-GFP expression in the trachea is suppressed in DreddD55; PGRP-LFKO double mutant larvae. (TIF) [file pgen.1006569.s004.tif]

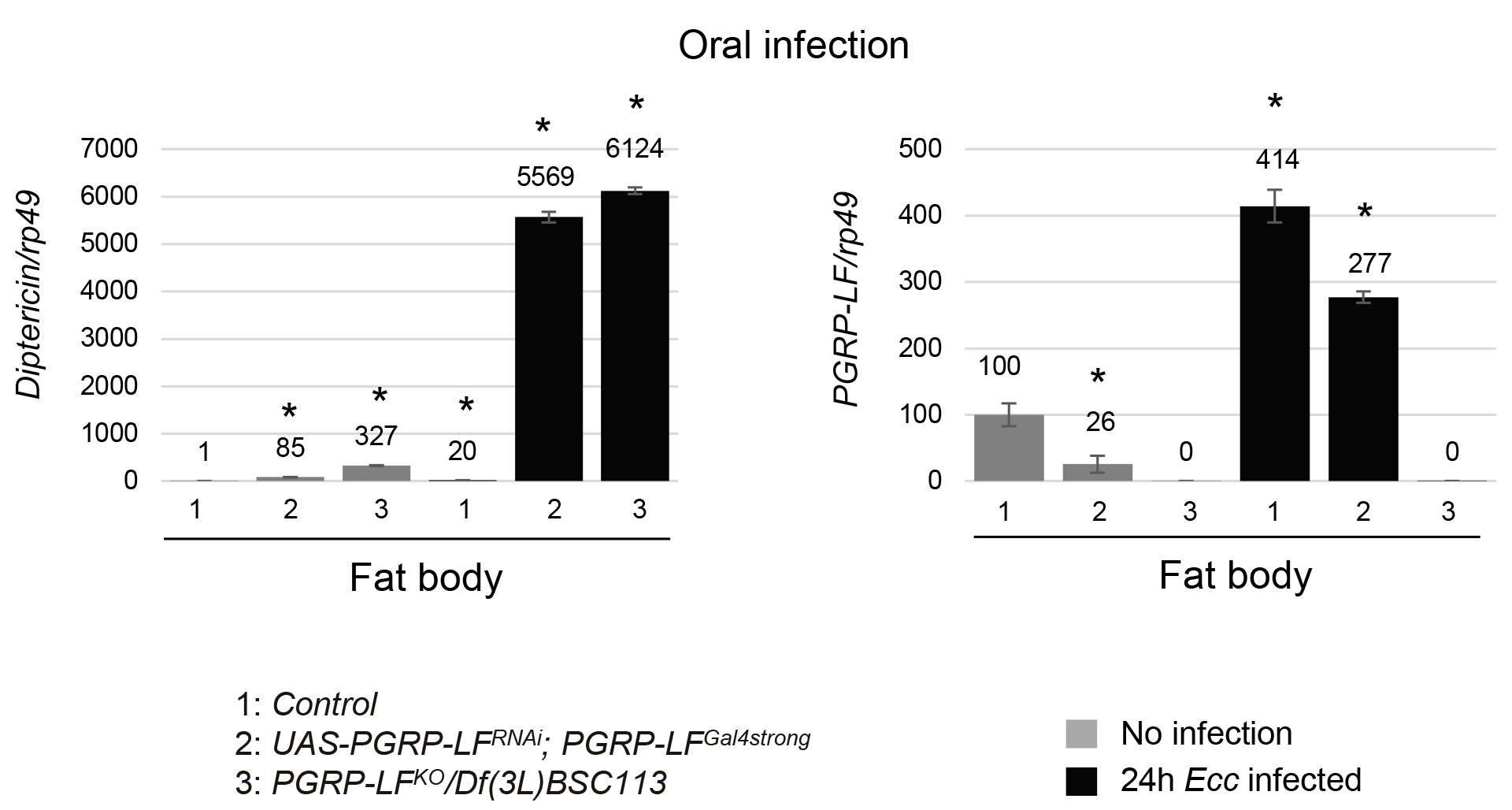

Supplement: S5 Fig — Fat body IMD pathway activation, monitored by Diptericin expression, 24h after Ecc oral infection. PGRP-LF inactivation by RNAi (PGRP-LFGal4strong; UAS-PGRP-LFRNAi/UAS-Dicer2) modifies IMD pathway inducibility in the fat body of Ecc orally infected flies as observed following complete inactivation of PGRP-LF in PGRP-LFKO/Df(3L)BSC113 flies. mRNA level in controls was set to 1, and values obtained with indicated genotypes were expressed as a fold of this value. Histograms correspond to the mean value ± SD of three independent experiments. Values indicated by symbols (*) are statistically significant (t-test, p < 0.05). ns: not significantly different. (TIF) [file pgen.1006569.s005.tif]

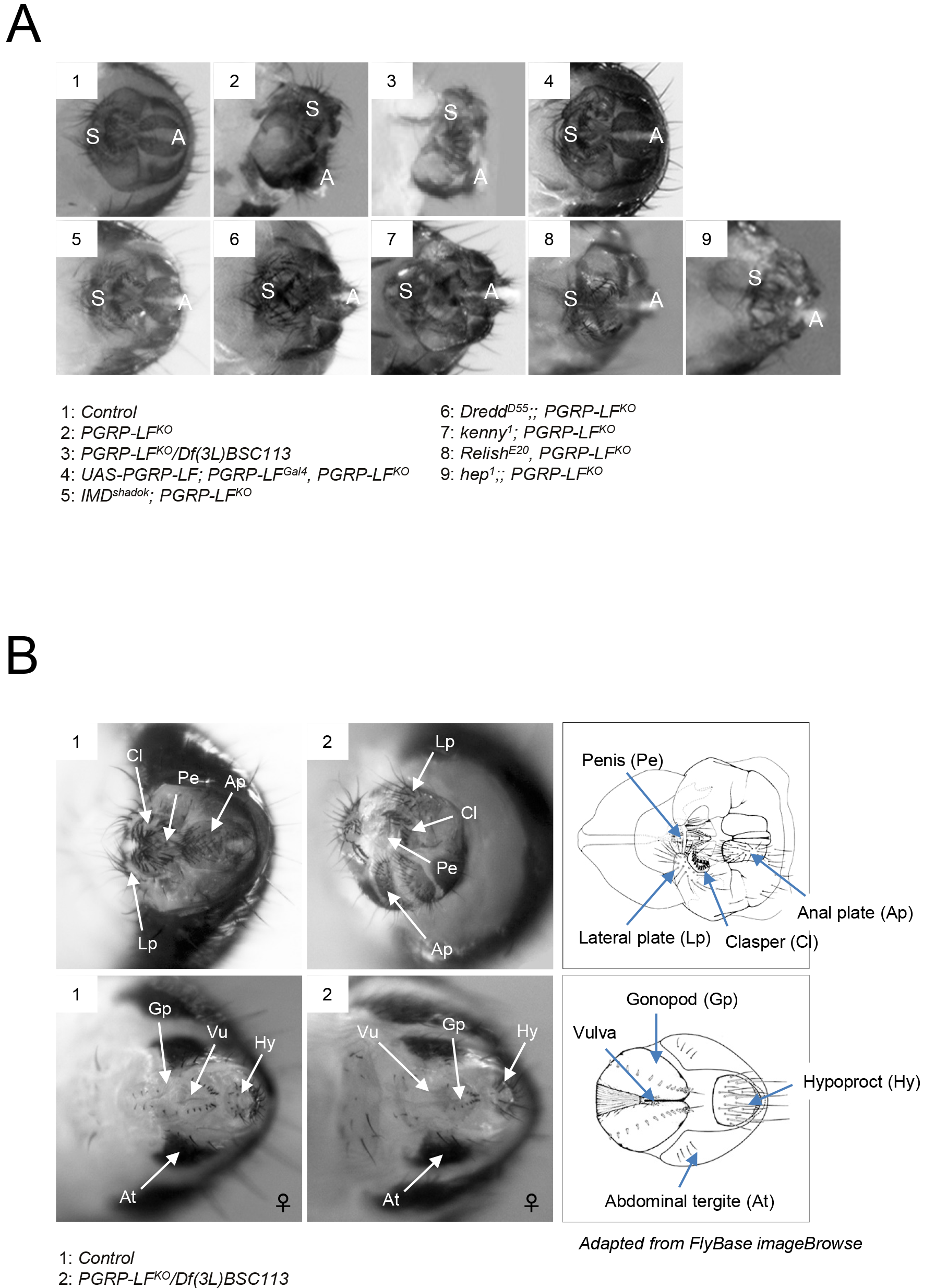

Supplement: S6 Fig — (A) Magnification of the pictures shown in Fig 5A. A and S indicate respectively anus and sexe primordia locations. (B) The external anatomy of both the male and female genitalia are not affected by the PGRP-LF mutation. (TIF) [file pgen.1006569.s006.tif]

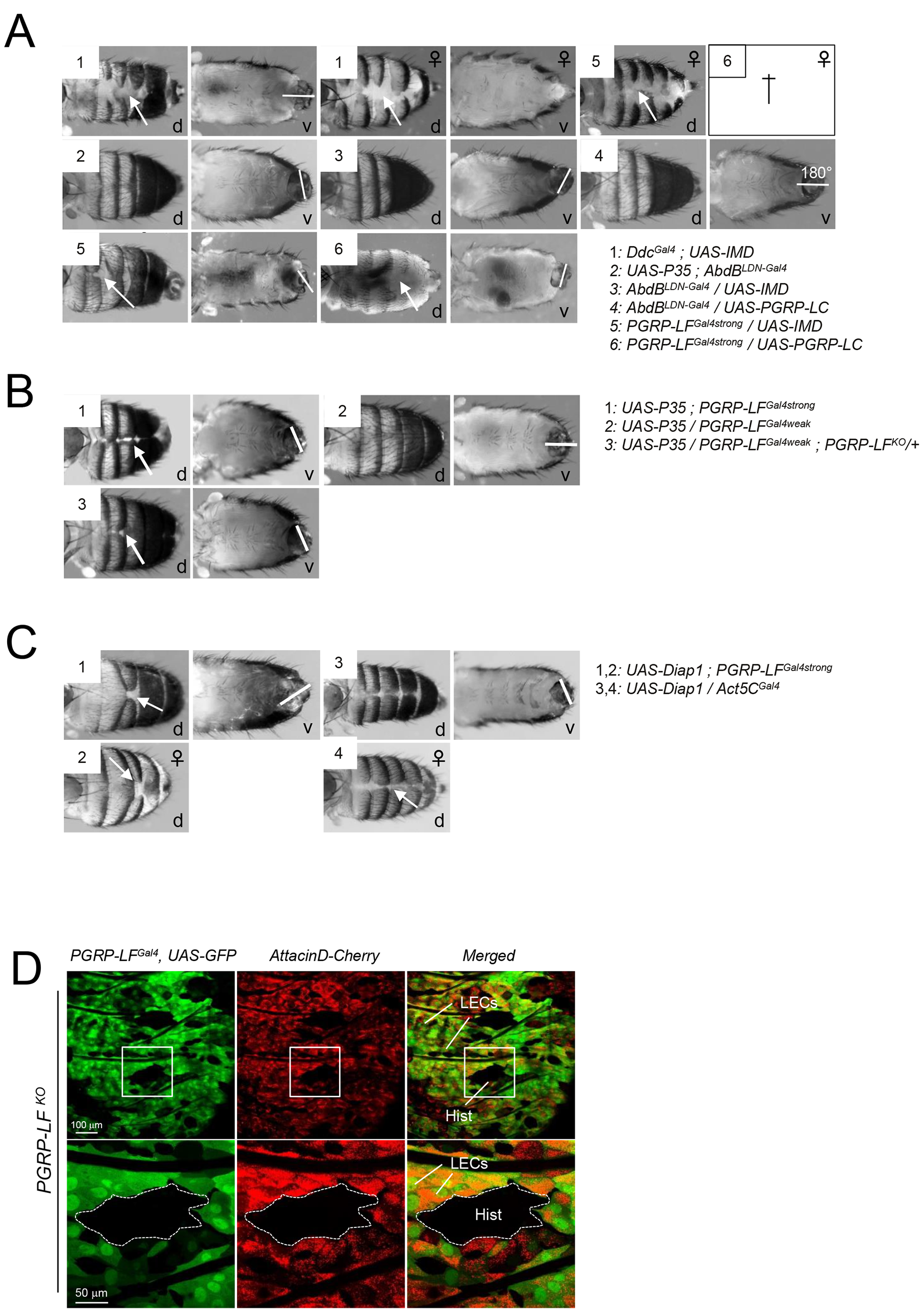

Supplement: S7 Fig — (A-C) Dorsal (d) and ventral (v) views of male or female adult abdomen showing incomplete epidermis differentiation (white arrow) and abnormal male genitalia orientation (white bar). (A) 1, 2: Overexpression of IMD in LECs with Ddc-Gal4 prevents normal dorsal epidermis fusion (arrow). Abnormal male genitalia orientation in flies overexpressing (3) P35, (4) IMD or (5) PGRP-LCa in AbdB expressing cells (white bar). Abnormal genitalia rotation and incomplete epidermis differentiation are both observed in PGRP-LFGal4strong / UAS-IMD or PGRP-LFGal4strong / UAS-PGRP-LCa flies. (B) Abdomen from adults overexpressing P35 under the control of (1) PGRP-LFGal4strong or (2 and 3) PGRP-LFGal4weak drivers. Incomplete fusion of dorsal epidermis and abnormal genitalia rotation are observed with PGRP-LFGal4strong or with PGRP-LFGal4weak when flies lack one copy of PGRP-LF gene. (C) Abdomen from adults overexpressing Diap1 under the control of (1 and 2) PGRP-LFGal4strong or (3 and 4) Act5CGal4 drivers. (D) Lateral views of PGRP-LFKO pupal cases expressing PGRP-LFGal4; UAS-nlsGFP and AttacinD-Cherry 24h APF. PGRP-LFGal4 and AttacinD-Cherry expression are both restricted to LECs and absent from histoblasts. The dashed lines indicate the boundary between the histoblasts and LECs. (TIF) [file pgen.1006569.s007.tif]

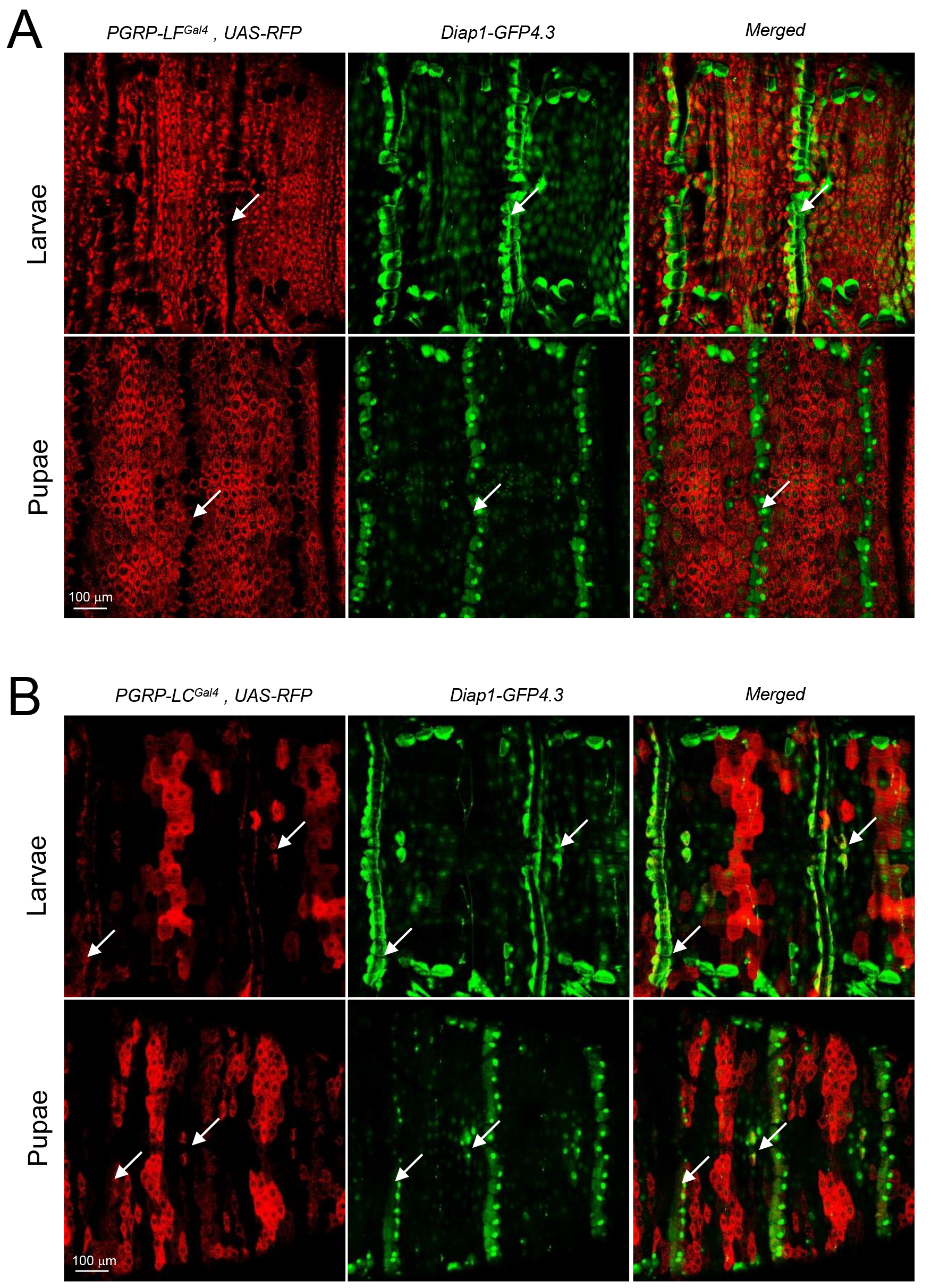

Supplement: S8 Fig — Dorsal view of Diap1-GFP4.3; PGRP-LFGal4, UAS-mcd8CherryRFP (A) or Diap1-GFP4.3; PGRP-LCGal4, UAS-mcd8-CherryRFP (B) third instar larvae or pupae 24h APF. (A) PGRP-LFGal4 is expressed in almost all dorsal LECs except in cells expressing strongly Diap1-GFP4.3 (arrows in A). (B) PGRP-LCGal4 is expressed in some dorsal LECs and is co-expressed with cells expressing strongly Diap1-GFP4.3 (arrows in B). (TIF) [file pgen.1006569.s008.tif]

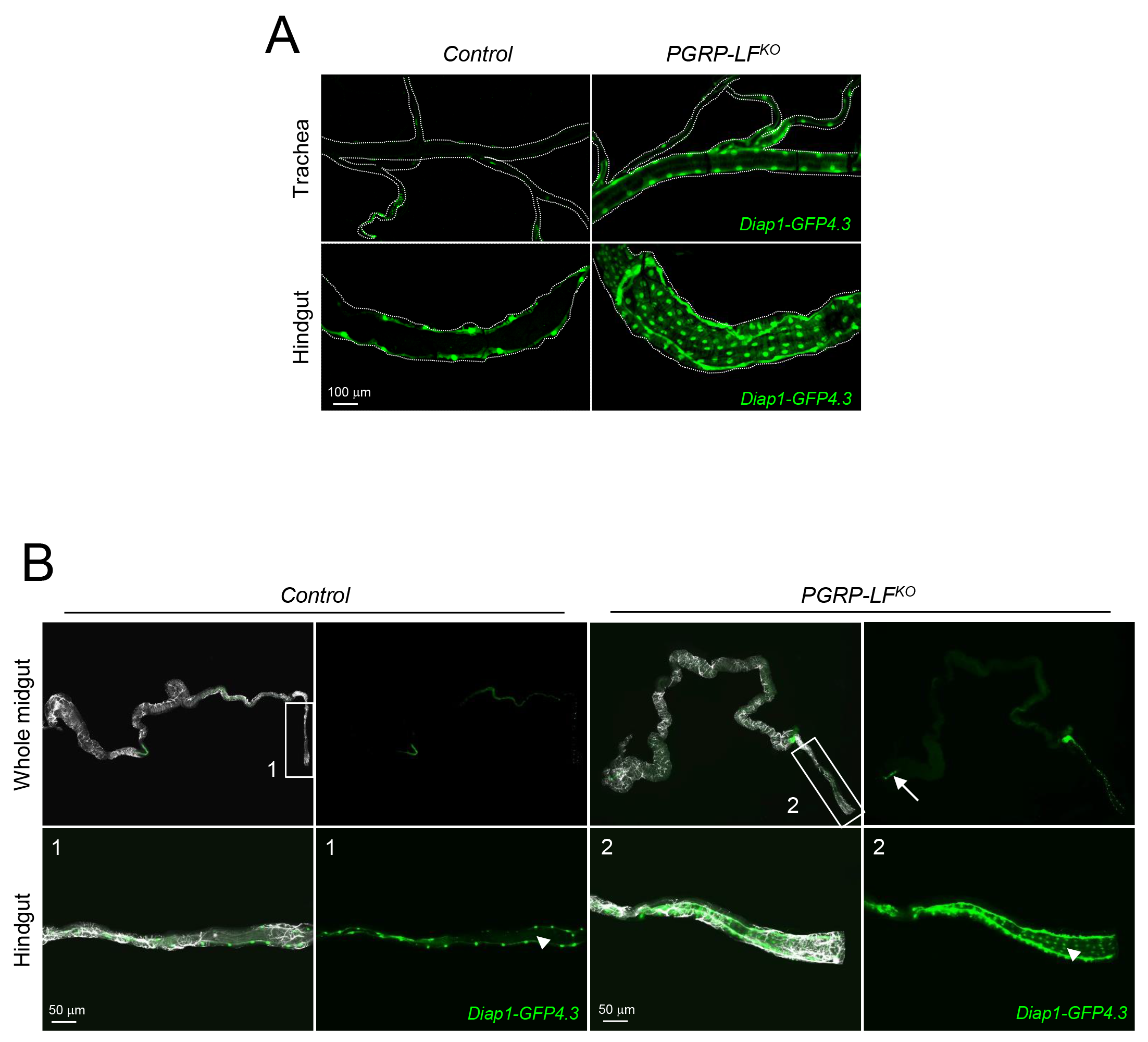

Supplement: S9 Fig — (A) Confocal images of dissected larval trachea and hindgut showing enhanced expression of Diap1-GFP4.3 in PGRP-LF mutant tissue when compared to controls. The dashed lines indicate the periphery of the control tissues. (B) Diap1-GFP4.3 is ectopically expressed in ectodermal derivatives of the larval gut of PGRP-LF mutants. Both the foregut (arrow) and the hindgut enterocytes (arrow head) express Diap1-GFP4.3 in PGRP-LF mutants compare to controls. (TIF) [file pgen.1006569.s009.tif]

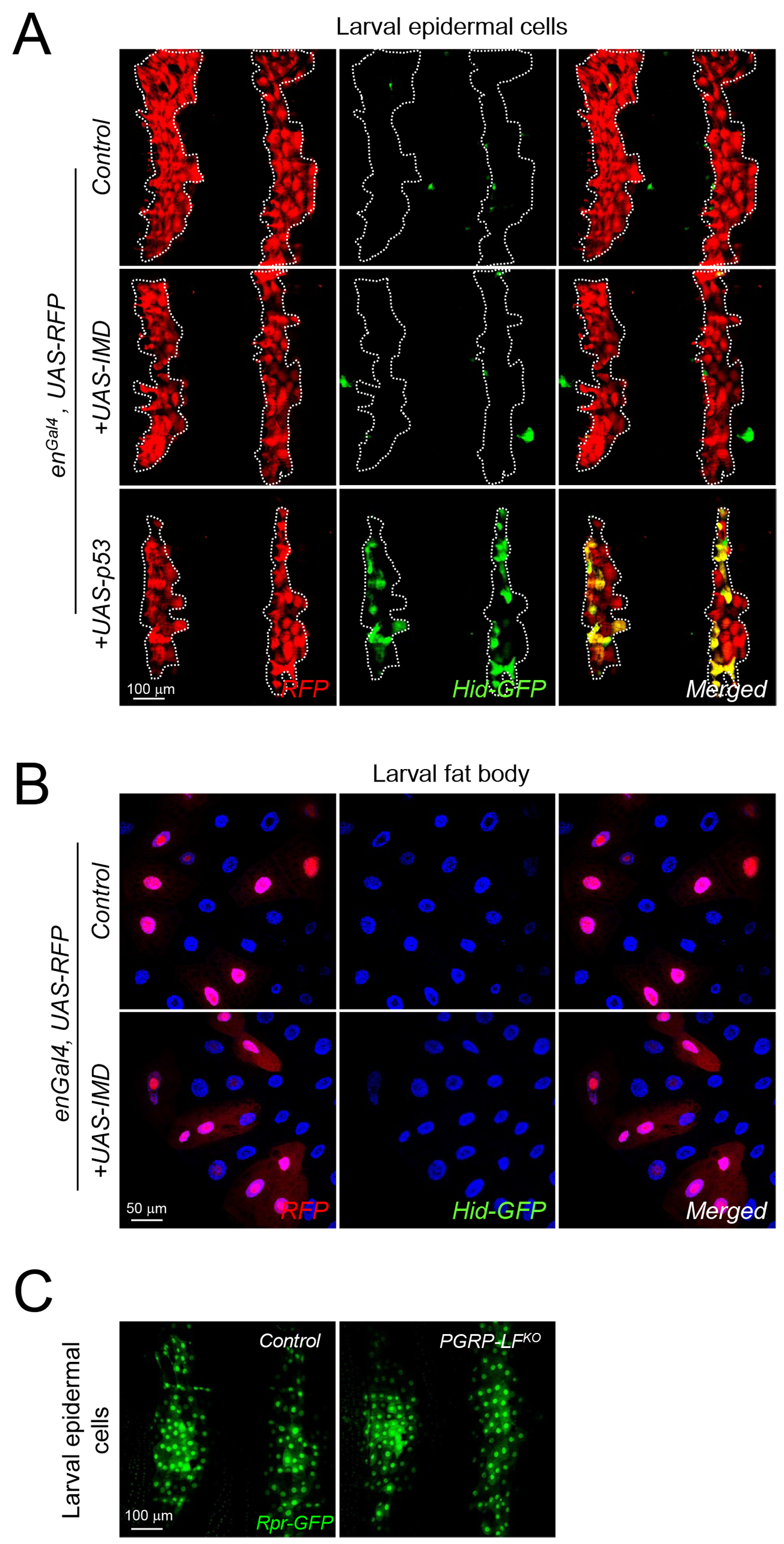

Supplement: S10 Fig — Hid-GFP expression is not induced in cells overexpressing IMD. Dorsal epidermis (A) and fat body (B) of enGal4, UAS-RFP/+ (control) or enGal4, UAS-RFP/+; UAS-IMD/+ (UAS-IMD) larvae are shown. In both LECs and fat body cells, expressing IMD do not induce expression of Diap1-GFP4.3 while in LECs p53 overexpression does (arrows). (C) ReaperGal4; UAS-nlsGFP expression is not ectopically expressed in LECs from third instar PGRP-LF mutant larvae. (TIF) [file pgen.1006569.s010.tif]

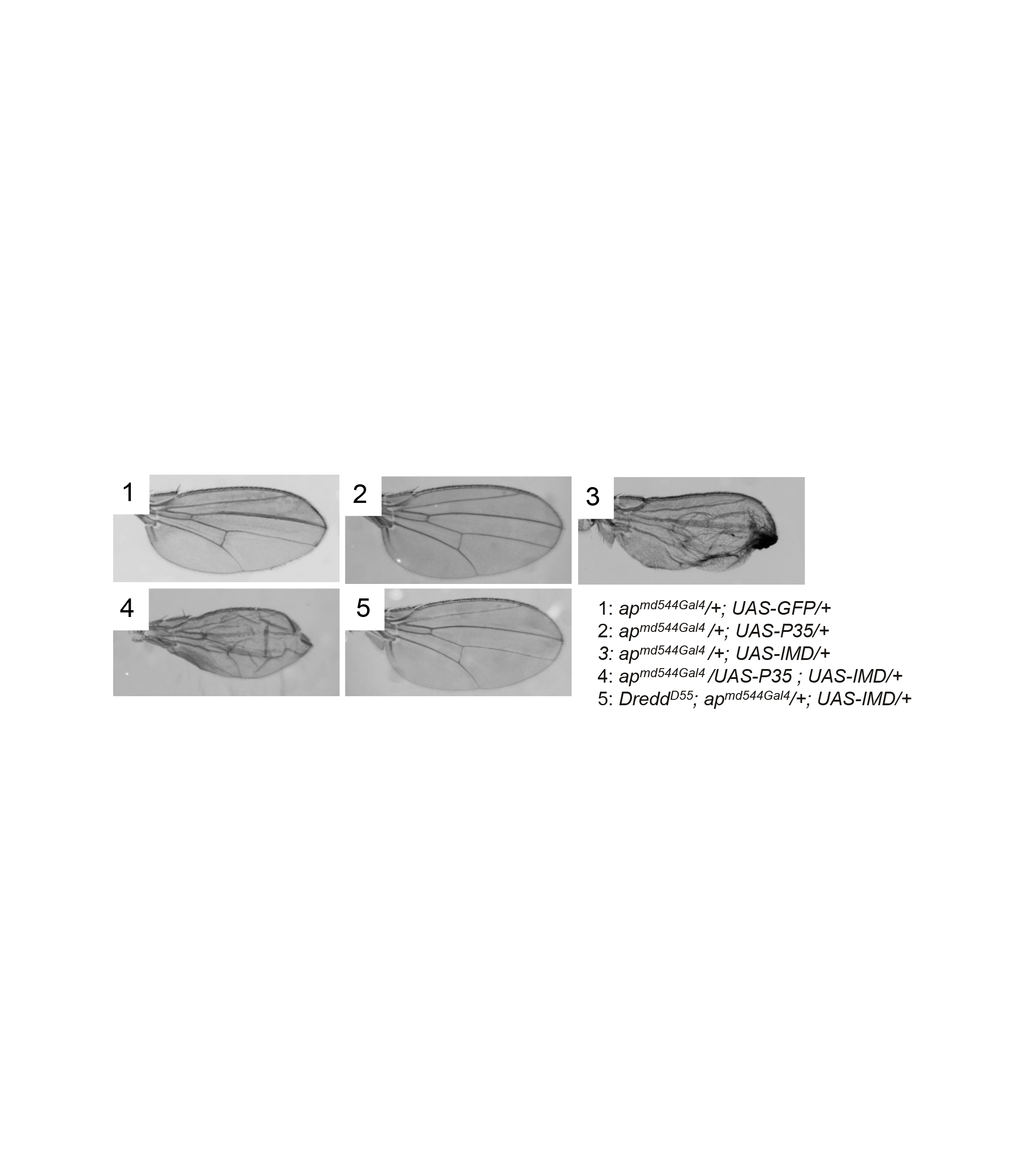

Supplement: S11 Fig — Adult wings from 5d old males of indicated genotype. Overexpression of IMD in the dorsal part of the wing pouch using apGal4 driver induces wing bubbles, a phenotype that is not suppressed by co-expression of the anti-apoptotic protein P35. (TIF) [file pgen.1006569.s011.tif]

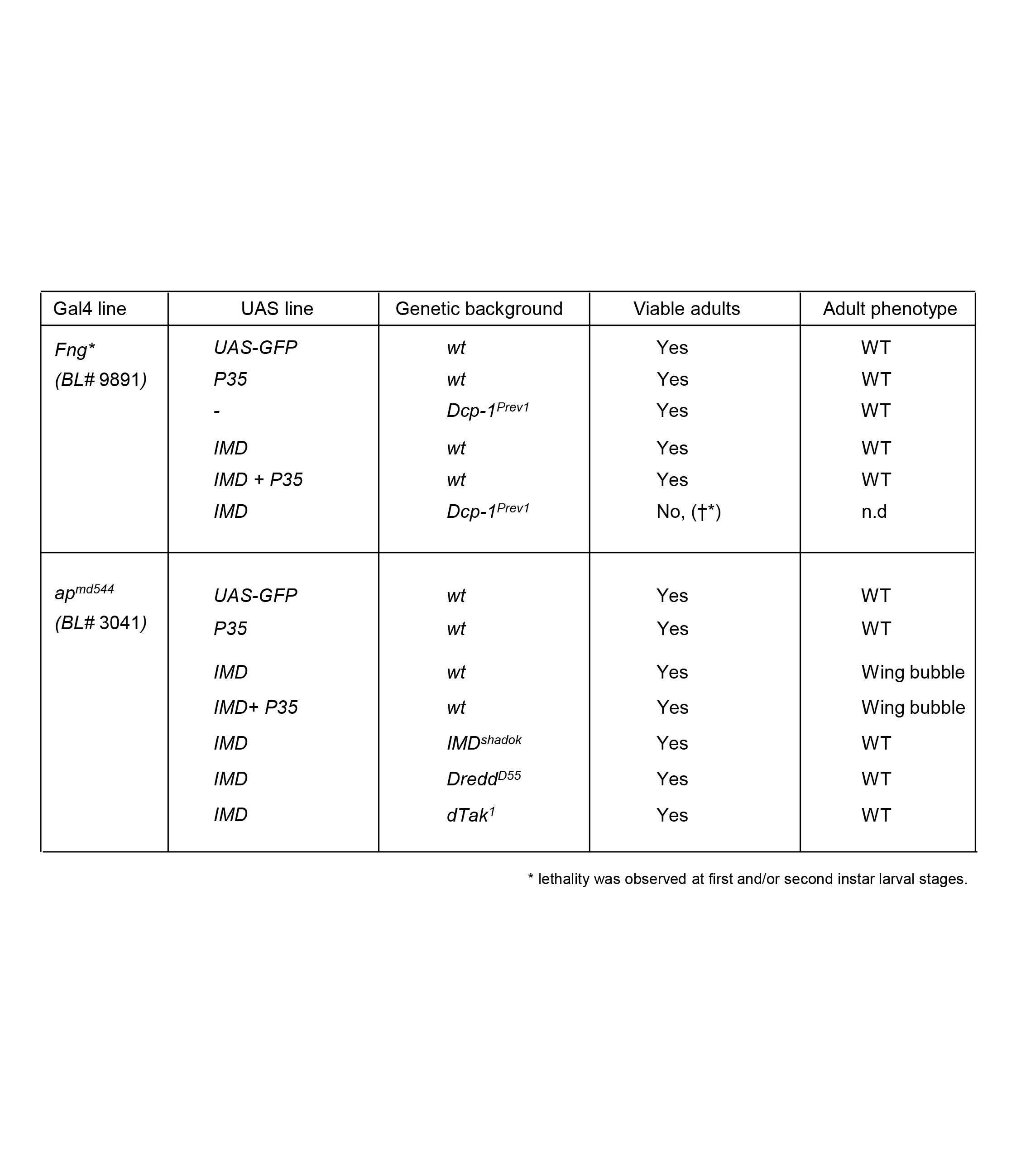

Supplement: S1 Table — Genetic interactions showing that lethality due to IMD gain-of-function is not suppressed, but rather enhanced, when apoptosis is blocked. (TIF) [file pgen.1006569.s012.tif]
